# Structures of the human pre-catalytic spliceosome and its precursor spliceosome

**DOI:** 10.1038/s41422-018-0094-7

**Published:** 2018-10-12

**Authors:** Xiechao Zhan, Chuangye Yan, Xiaofeng Zhang, Jianlin Lei, Yigong Shi

**Affiliations:** 1 Beijing Advanced Innovation Center for Structural Biology, Tsinghua-Peking Joint Center for Life Sciences, School of Life Sciences and School of Medicine, Tsinghua University, Beijing, 100084 China; 2 Technology Center for Protein Sciences, Ministry of Education Key Laboratory of Protein Sciences, School of Life Sciences, Tsinghua University, Beijing, 100084 China; 3 Institute of Biology, Westlake Institute for Advanced Study, Westlake University, 18 Shilongshan Road, Xihu District, Hangzhou, Zhejiang 310024 China

## Abstract

The pre-catalytic spliceosome (B complex) is preceded by its precursor spliceosome (pre-B complex) and followed by the activated spliceosome (B^act^ complex). The pre-B-to-B and B-to-B^act^ transitions are driven by the ATPase/helicases Prp28 and Brr2, respectively. In this study, we report the cryo-electron microscopy structures of the human pre-B complex and the human B complex at an average resolution of 5.7 and 3.8 Å, respectively. In the pre-B complex, U1 and U2 small nuclear ribonucleoproteins (snRNPs) associate with two edges of the tetrahedron-shaped U4/U6.U5 tri-snRNP. The pre-mRNA is yet to be recognized by U5 or U6 small nuclear RNA (snRNA), and loop I of U5 snRNA remains unengaged. In the B complex, U1 snRNP and Prp28 are dissociated, the 5’-exon is anchored to loop I of U5 snRNA, and the 5′-splice site is recognized by U6 snRNA through duplex formation. In sharp contrast to *S. cerevisiae*, most components of U2 snRNP and tri-snRNP, exemplified by Brr2, undergo pronounced rearrangements in the human pre-B-to-B transition. Structural analysis reveals mechanistic insights into the assembly and activation of the human spliceosome.

## Introduction

Pre-mRNA splicing is executed by the spliceosome, which undergoes an ordered process of assembly and activation prior to catalysis.^1,2^ The pre-mRNA is first recognized by U1 snRNP, forming an early spliceosome (E complex), and then by U2 snRNP, becoming the pre-spliceosome (A complex). In the A complex, the 5’-splice site (5’SS) and the branch point sequence (BPS) are recognized by U1 and U2 snRNPs, respectively, involving duplex formation with conserved sequences of U1 and U2 snRNAs^2^. The A complex associates with the U4/U6.U5 tri-snRNP to generate the pre-B complex. The pre-B complex undergoes RNP remodeling to become the B complex, which is converted into the activated spliceosome (B^act^ complex).^3^ The pre-B-to-B and B-to-B^act^ transitions are mediated by the RNA-dependent ATPase/helicases Prp28 and Brr2, respectively.^4,5^ Beyond the B^act^ complex, the spliceosome sequentially adopts five distinct compositional states: the catalytically activated spliceosome (B^*^ complex, where branching occurs), catalytic step I spliceosome (C complex), step II catalytically activated spliceosome (C^*^ complex, where exon ligation occurs), post-catalytic spliceosome (P complex), and intron lariat spliceosome (ILS complex). The B^*^-to-C and C^*^-to-P transitions proceed spontaneously, whereas the other three transitions (B^act^-to-B^*^, C-to-C^*^, and P-to-ILS) are also driven by other ATPase/helicases.^4,5^

Compared to that in yeast, the human spliceosome contains more regulatory components and has a considerably more dynamic conformation, both of which pose challenges for structural characterization. Despite rigorous effort, only four states of the assembled human spliceosome have been structurally captured at moderate resolutions: B,^6^ B^act^^7,8^ C,^9^ and C^*^^10,11^. Among the other four states of the human spliceosome, the pre-B complex is of particular note, because its existence was suggested 2 years ago from the biochemical finding that a compositionally distinct state may populate prior to the B complex in the absence of a functional Prp28.^12^ This conjecture is supported by structural comparison of the B complex with the U4/U6.U5 tri-snRNP.^6,13^ In addition, the pre-B complex, which bridges the spliceosomal A and B complexes, represents the first and only assembled spliceosome where all five snRNPs are present.

In this study, we report the cryo-electron microscopy (cryo-EM) structures of the entire human pre-B complex at an average resolution of 5.7 Å and the human B complex at 3.8 Å (Supplementary information, Tables S1–S3). These structures uncover a number of unique and functionally important features in both the pre-B and the B complexes. Comparison between the human pre-B and B complexes reveals the impact of U1 snRNP and Prp28 dissociation. Notably, the pre-B-to-B transition in human exhibits major differences compared to that in *S. cerevisiae.*^14^ This study fills an important void in the structural characterization of the human spliceosome and allows mechanistic understanding of its assembly and activation.

## Results

### Spliceosome isolation and electron microscopy

Human spliceosomes were in vitro assembled using the MINX pre-mRNA that contains three tandem MS2 sites at the 3’-end of the 3’-exon. Because reversible protein phosphorylation is indispensable for pre-mRNA splicing,^15,16^ we were able to prevent splicing using phosphatase inhibitors, arresting the spliceosome in its earlier states (Supplementary information, Fig. S1a, b). The spliceosomes were affinity-purified and glutaraldehyde-crosslinked during glycerol gradient centrifugation (Supplementary information, Fig. S1b). The structural integrity of the spliceosome was examined using the negative staining EM (Supplementary information, Fig. S1c). Cryo-EM samples were imaged on a Titan Krios electron microscope (FEI Company) using a Gatan K2 Summit detector (Gatan Company) (Supplementary information, Fig. S1d).

1.42 million particles were auto-picked and subjected to a guided multi-reference classification procedure as described previously^9^ (Supplementary information, Fig. S2). After global classification, particles that belong to the pre-B or B complex were separately treated as the input for subsequent local classifications. Following two rounds of multi-reference three-dimensional (3D) classification, 186,161 particles yielded a reconstruction of the entire human pre-B complex at an average resolution of 5.7 Å on the basis of the FSC value 0.143 (Supplementary information, Figs. S2 & S3a, Table S1). A similar procedure on the human B complex allowed selection of 137,853 particles, which produced a structure at an average resolution of 3.8 Å (Supplementary information, Figs. S2 & S3a, Table S1). The local resolution reaches 4.5 and 3.0 Å in the core of the pre-B and B complexes, respectively (Supplementary information, Fig. S3). The resulting EM density maps display distinguishing features for the proteins and the RNA elements (Supplementary information, Figs. S4–S7). These maps allow determination of the secondary structural elements in the core regions of both complexes and assignment of specific sequences in the core of the B complex. The modeling was facilitated by the structures of the human tri-snRNP,^13^ the B complex,^6^ the B^act^ complex,^8^ and the C^*^ complex^10^ (Supplementary information, Tables S2 & S3).

### Overall structure of the human pre-B complex

**Fig. 1:**
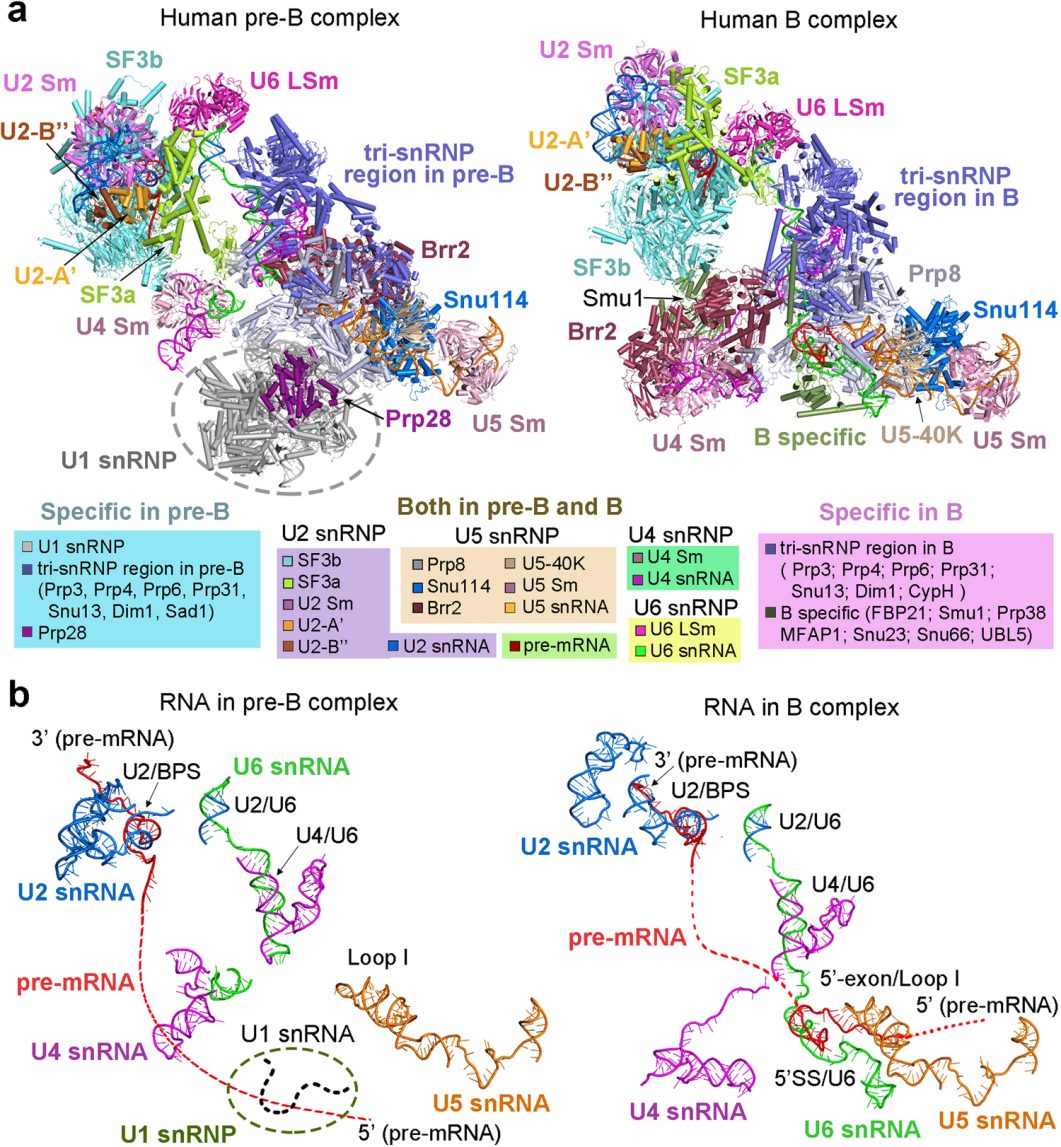
Cryo-EM structures of the human precursor pre-catalytic spliceosome (the pre-B complex) and the pre-catalytic spliceosome (the B complex). **a** Structures of the pre-B complex (left panel) and the B complex (right panel). The protein and RNA components are color-coded and tabulated below the images. The pre-B complex, but not the B complex, contains Sad1, Prp28, and U1 snRNP. Components of U1 snRNP (colored grey) remain unassigned due to the low resolution of the EM density map in this region. Compared to pre-B, the B complex has eight additional protein components: CypH, FBP21, MFAP1, Prp38, Smu1, Snu23, Snu66, and UBL5. Although the U4/U6.U5 tri-snRNP is the core in both complexes, it undergoes pronounced structural rearrangement. **b** Structures of the RNA elements in the pre-B complex (left panel) and the B complex (right panel). The pre-mRNA, U2, U5, and U6 snRNAs are colored red, marine, orange, and green, respectively. This color scheme is preserved throughout this manuscript. In the pre-B complex, the BPS is already recognized by complementary sequences of U2 snRNA through duplex formation, and the 3’-end sequences of U6 snRNA forms helix II with the 5’-end sequences of U2 snRNA. The 5’-exon of the pre-mRNA is anchored to loop I of U5 snRNA in the B complex, but not the pre-B complex. All structural images were created using PyMol^68^

The human pre-B complex comprises U1 snRNP, U2 snRNP, and the centrally located U4/U6.U5 tri-snRNP, which are loosely bound together to yield a highly asymmetric and dynamic assembly (Fig. 1a, left panel). Both U1 and U2 snRNPs appear to be flexibly connected to the tri-snRNP, as judged by the features of their EM density maps (Supplementary information, Fig. S4a–d). The tri-snRNP has the appearance of an irregular tetrahedron, with its four corners corresponding to the U4 Sm ring, the U5 Sm ring, the U6 LSm ring, and Brr2 (Fig. 1a, left panel). U2 snRNP is held on one edge of the tetrahedron between the U4 Sm ring and the U6 LSm ring, whereas U1 snRNP is positioned along the neighboring edge between the U4 and U5 Sm rings. U1 and U2 snRNPs do not interact with each other and are separated by a gap of 60–100 Å; one corner of the tri-snRNP tetrahedron—the U4 Sm ring and the 3’-end sequences of U4 snRNA (nucleotides 83–145)—is located in this gap. The far ends of U1 and U2 snRNPs span a distance of over 390 Å. For the pre-B complex, the distances exceed 400 and 370 Å, respectively, from the U2 Sm ring to Brr2 and to the U5 Sm ring.

The U4/U6.U5 tri-snRNP serves as the stable core of the pre-B complex and is well defined by the EM density (Supplementary information, Fig. S4d). The structures and locations of all protein and RNA components of the tri-snRNP in the pre-B complex are nearly identical to those in the isolated human tri-snRNP^13^ but differ significantly to those in the B complex^6^ (Fig. 1a, right panel). U2 snRNP, which comprises the SF3a and SF3b complexes and the core components (U2 snRNA, the Sm ring, and U2-A’ and U2-B’’), is well defined in the EM map and is connected to the tri-snRNP mainly through interactions between the SF3b complex and the heptameric U6 LSm ring (Supplementary information, Fig. S4a, b). Compared to U2 snRNP, U1 snRNP is more flexibly connected to the tri-snRNP and can only be located into the current EM density map without a precise orientation (Supplementary information, Fig. S4a, c). The placement of U1 snRNP is supported by both the large size and the location of the EM density lobe, which is connected to the ATPase/helicase Prp28 (Supplementary information, Fig. S4c, d). In contrast to *S. cerevisiae,*^14^ Prp28 is unambiguously identified on the edge of the tri-snRNP between the U4 and U5 Sm rings; such a location presumably allows it to conveniently dissociate the neighboring U1 snRNP from the 5’SS.^17–19^

### Structural comparison with the human B complex

Structural analysis reveals a rigid core between the pre-B and B complexes that maintains the same general conformation; this core includes the entire U5 snRNA, the N-domain of Prp8, Snu114, U5-40K, and the U5 Sm ring. All subsequent discussions on the structural changes during the pre-B-to-B transition are based on alignment of this core. Compared to the pre-B complex, the EM density that corresponds to U1 snRNP has largely disappeared in the B complex^6^ (Supplementary information, Fig. S5), indicating its dissociation in the pre-B-to-B transition. In addition, a number of the protein components in the tri-snRNP have been translocated in the pre-B-to-B transition (Fig. 1a). Most notably, Brr2, which drives the B-to-B^act^ transition,^20,21^ is shifted from one corner of the tri-snRNP in the pre-B complex to the same corner as occupied by the U4 Sm ring in the B complex^6^ (Fig. 1a, right panel). The movement of Brr2 changes the overall appearance of the tri-snRNP from a tetrahedron to a planar triangle. The entire SF3a and SF3b complexes have undergone a 60-degree rotation in the pre-B-to-B transition, positioning the SF3b complex in direct contact with the B complex specific protein Smu1 (Fig. 1a, right panel).

Concurrent with the drastic changes of the overall structure, the RNA elements exhibit major differences between the pre-B and B complexes (Fig. 1b; Supplementary information, Fig. S6). In the pre-B complex, the BPS and surrounding nucleotides of the intron are recognized by the conserved sequences (nucleotides 31–45) of U2 snRNA through duplex formation, and the 3’-end sequences of U6 snRNA already form helix II with the 5’-end sequences of U2 snRNA^12,22,23^ (Fig. 1b, left panel; Supplementary information, Fig. S6a). The 5’SS is presumably recognized by U1 snRNA in the pre-B complex, although this feature is not resolved in the current EM density map. Neither U5 nor U6 snRNA directly engages the pre-mRNA in the pre-B complex. In the B complex,^6^ however, the 3’-end sequences of the 5’-exon are already anchored to loop I of U5 snRNA and the ensuing 5’SS forms a duplex with the ACAGA box of U6 snRNA^24–26^ (Fig. 1b, right panel; Supplementary information, Fig. S6b). The 3’-end sequences of U4 snRNA and the 5’-end sequences of U6 snRNA have been rearranged to allow the engagement of pre-mRNA in the B complex.^6^ Notably, U5 snRNA, helix II of the U2/U6 duplex, the U4/U6 duplex, and the 5’-end sequences of U4 snRNA remain largely static in the pre-B-to-B transition.

### Major changes around loop I of U5 snRNA

**Fig. 2:**
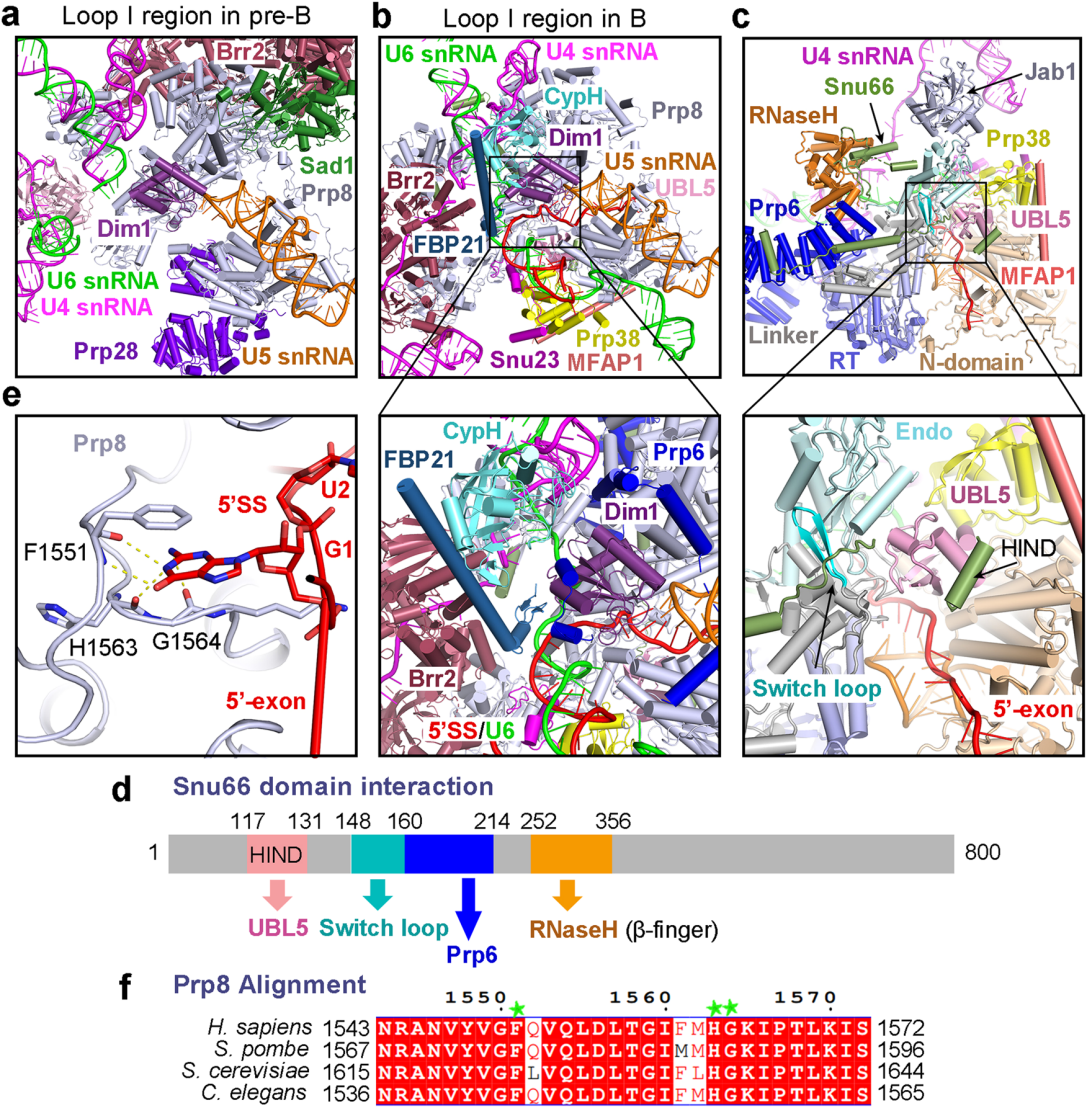
Structural changes around loop I of U5 snRNA in the pre-B-to-B transition. **a** Structure of the proteins and RNA elements in the core of the human pre-B complex. Dim1 directly contacts the tip of the U5 loop I, which remains unoccupied. U6 snRNA is yet to engage the 5’SS. The ATPase/helicase Prp28 binds to the N-domain of Prp8. **b** Structure of the proteins and RNA elements in the core of the human B complex. The structure here is displayed in the same orientation as that of the pre-B complex in panel A. In contrast to the pre-B complex, Prp28 is absent and the 5’-exon sequences of the pre-mRNA are anchored to loop I of U5 snRNA. Three proteins (MFAP1, Prp38, and Snu23) have been recruited to the general location previously occupied by Prp28. Prp38 and Snu23 interact with the newly formed U6/5’SS duplex. FBP21 closely associates with CypH, which together binds neighboring proteins and the U6/5’SS duplex. A close-up view is shown in the lower panel to highlight the FBP21-CypH subcomplex and its surrounding proteins and RNA elements. Brr2 undergoes a drastic translocation in the transition from the pre-B to the B complex. Dim1 remains unchanged in the transition. **c** Structures of the B complex specific protein Snu66 and UBL5. Snu66 assumes an extended conformation in the human B complex and interacts with UBL5, Prp6, and the Linker and RNaseH-like domain of Prp8. A close-up view is shown in the lower panel to highlight UBL5 and its surrounding components. **d** A cartoon diagram of Snu66 and its binding elements. Snu66 interacts with UBL5, Prp6, the Switch loop and the β-finger of Prp8. **e** A close-up view on the recognition of the 5’SS. The guanine nucleobase of G1 in the 5’SS stacks against Phe1551 of Prp8 and makes hydrogen bonds (H-bonds) to the main chain atoms of Phe1551, His1563, and Gly1564. **f** The amino acid sequences of Prp8 that recognize the 5’SS are highly conserved across species. The primary sequences of human Prp8 are aligned with those of the Prp8 orthologues from yeast (*S. pombe* and *S. cerevisiae*) and worm (*C. elegans*). Residues that are involved in the recognition of G1 of the 5’SS are marked by green asterisks

U5 snRNA is the center of the rigid core shared by the pre-B and B complexes; its conformation is mainly sustained by the N-domain of Prp8, Snu114, U5-40K, and the heptameric Sm ring. In the pre-B complex, the small protein Dim1 directly interacts with the tip of the U5 loop I, which remains unoccupied (Fig. 2a; Supplementary information, Fig. S7a). Dim1 is oriented mainly by the Thumb/X, the Linker, and a portion of the N-domain (residues 663–798) of Prp8 (Supplementary information, Fig. S8a). Notably, the N-terminal fragment (residues 4–26) of the tri-snRNP-specific protein Prp6 forms two short α-helices on the exposed surface of Dim1. Sad1 bridges the interaction between the RT Fingers/Palm of Prp8 and the PWI domain of Brr2 (Fig. 2a; Supplementary information, Fig. S8a). The ATPase/helicase Prp28 interacts with the N-domain of Prp8 while binding to U1 snRNP.

During the pre-B-to-B transition, Sad1, Prp28, and U1 snRNP are dissociated, and at least eight proteins (CypH, FBP21, MFAP1, Prp38, Smu1, Snu23, Snu66, and UBL5) are recruited into the B complex.^6^ Similar to that in the pre-B complex, Dim1 in the B complex interacts with the N-terminal α-helices of Prp6 and remains registered with respect to a portion of the N-domain (residues 663–798), Thumb/X, and Linker domains of Prp8 (Fig. 2b, inset; Supplementary information, Fig. S8b). In the B complex,^6^ loop I of U5 snRNA already forms a duplex with the 5’-exon sequences (Supplementary information, Fig. S7b, f), which are followed by an extended duplex involving the ensuing 5’SS and the ACAGA box of U6 snRNA.^24–26^ Most notably, the single-stranded sequences of U4 snRNA are already loaded into the N-terminal cassette domain (NC) of Brr2^27^ (Fig. 2b).

The U6/5’SS duplex closely associates with the N-domain of Prp8 and the B complex specific proteins Snu23 and Prp38 (Fig. 2b; Supplementary information, Fig. S8b). Snu23, Prp38, and MFAP1 assemble into a stable ternary complex,^28^ of which the latter two proteins directly interact with the N-domain of Prp8. FBP21 and the peptidyl prolyl isomerase CypH closely associate with each other and stabilize the local conformation through direct interactions with multiple surrounding proteins. For FBP21, the N-terminal β-hairpin directly contacts Dim1, the ensuing short α-helix binds the U6/5’SS duplex, and the extended α-helix interacts with CypH and the NC of Brr2 (Fig. 2b, inset). These structural features are consistent with a role of FBP21 in the regulation of the Brr2 helicase activity.^29^ CypH simultaneously binds to Prp4 and Prp6.^30^

The B complex specific protein Snu66 in an extended conformation interacts with Prp6 and the Linker and RNaseH-like domains of Prp8; it also binds to ubiquitin like protein 5 (UBL5, equivalent of Hub1 in *S. cerevisiae*) through its single HIND element^31^ (Fig. 2c, d; Supplementary information, Fig. S7c, d). Most notably, two prominent surface loops from Snu66 appear to play an important role in the B complex by stabilizing two catalytic motifs—the Switch loop and the β-finger—of Prp8. One such loop (residues 148–158 of Snu66), located between an extended α-helix and the HIND element, hovers above the Switch loop and presumably blocks its potential movement (Fig. 2c, inset). Intriguingly, compared to the B complex, the Switch loop in the B^act^ complex and beyond is flipped by nearly 180 degrees to stabilize the bound 5’-exon sequences.^1^ The other surface loop closely interacts with the β-finger and stabilizes the RNaseH-like domain (Fig. 2c; Supplementary information, Fig. S7c). Therefore, Snu66 appears to function as a scaffold in the spliceosome prior to its activation. UBL5 directly interacts with the 5’-exon sequences of the 5’-exon/loop I duplex and is surrounded by Prp38 and the N-domain, Linker and endonuclease-like domain of Prp8 (Fig. 2c, inset; Supplementary information, Fig. S7d). These structural findings are consistent with the conclusion that UBL5 is a noncanonical splicing factor and may play an important role in alternative splicing.^31,32^

In the B complex, the pre-mRNA sequences immediately downstream of the 5’-exon are recognized by the Linker domain of Prp8^33^ (Fig. 2e, f; Supplementary information, Fig. S7e, f). The guanine base of the first nucleotide (G1) in the 5’SS stacks against the aromatic side chain of Phe1551 of Prp8 and makes putative hydrogen bonds (H-bonds) to the backbone amide and carbonyl groups of Phe1551, His1563, and Gly1564 of Prp8 (Fig. 2e; Supplementary information, Fig. S7e). These residues are invariant and the surrounding sequences are highly conserved in the Prp8 orthologues from yeast, worm, and human (Fig. 2f). Notably, the mode of recognition for G1 of the 5’SS is reminiscent of that in the yeast U4/U6.U5 tri-snRNP.^34^

### Structural rearrangements in Prp8 and Brr2

**Fig. 3:**
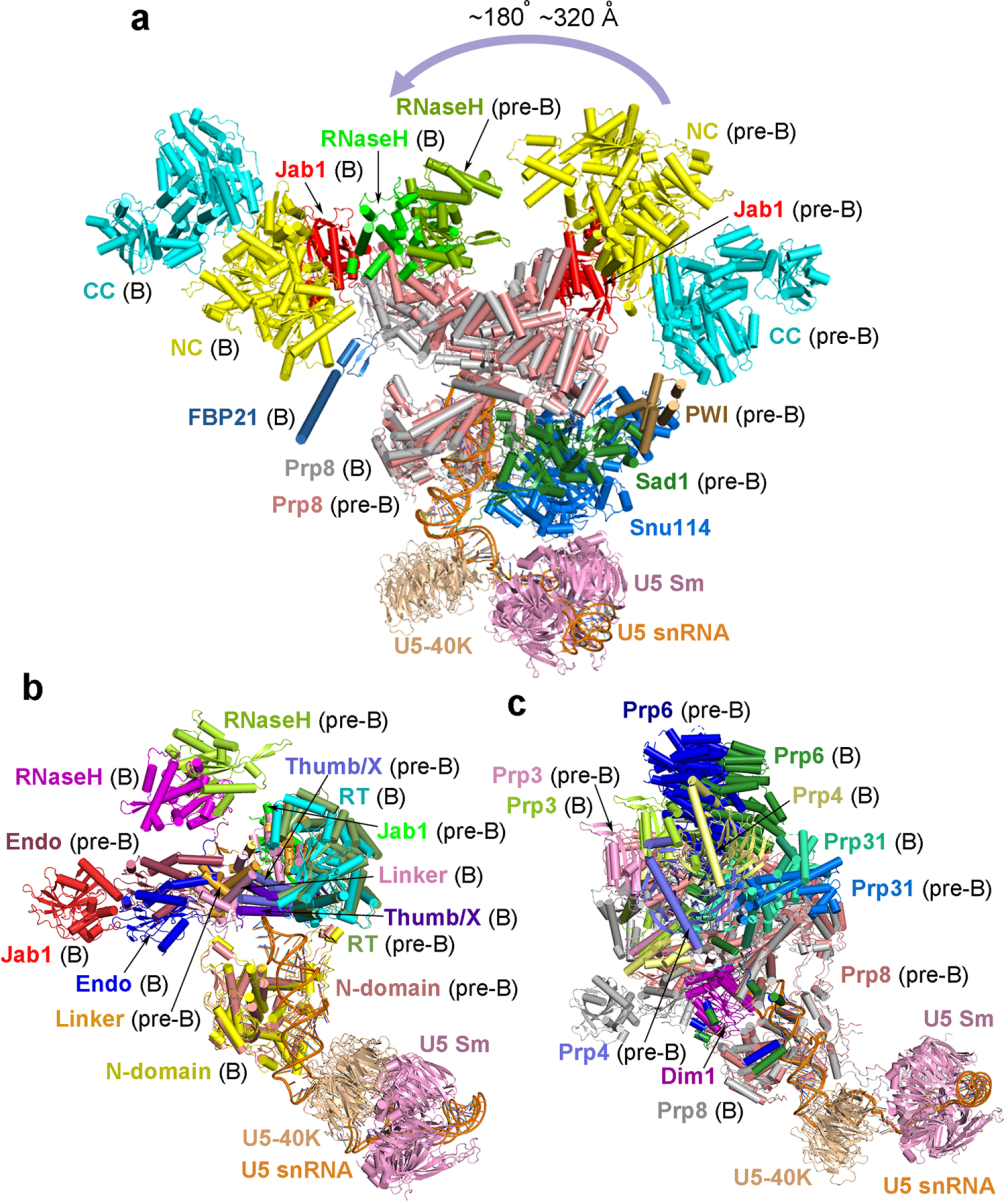
Structural comparison of the protein components between the human pre-B complex and the human B complex. **a** Structural changes of Brr2 and Prp8 in the pre-B-to-B transition. These changes are based on the alignment of U5 snRNA between the pre-B and B complexes. Brr2 is translocated by 120–320 Å and flipped by nearly 180 degrees. The Jab1 domain of Prp8, which stably associates with Brr2, is similarly translocated. The RNaseH-like domain of Prp8 is rotated by about 180 degrees. The four domains in the core of Prp8 (Fingers/Palm, Thumb/X, Linker, endonuclease-like) undergo varying degrees of positional shift. **b** A close-up view of Prp8 in the pre-B-to-B transition. Notably, the U5 Sm ring, U5-40K, and the N-domain of Prp8 remain largely unchanged. All other domains of Prp8 undergo noticeable structural rearrangements, which make the inter-domain organization of Prp8 more compact. **c** Structural changes of select proteins in the U4/U6.U5 tri-snRNP. Most proteins of the tri-snRNP undergo translocation and conformational changes in the pre-B-to-B transition. Highlighted here are Prp3, Prp4, Prp6, and Prp31, each of which undergoes pronounced translocation. The C-terminal fragment of Prp3 and the TPR repeats of Prp6 are shifted by approximately 25 and 35 Å, respectively

As the most conserved and largest component of all spliceosomal proteins, Prp8 anchors the active site RNA elements.^35–37^ Except its N-domain, all other domains of Prp8 undergo noticeable structural rearrangements in the pre-B-to-B transition. The C-terminal Jab1 domain, which is stably bound to Brr2,^38,39^ is translocated by 110–130 Å (Fig. 3a). The RNaseH-like domain is flipped by nearly 180 degrees, with the β-finger pointing to opposite directions in the pre-B and B complexes (Fig. 3b). The conserved core of Prp8 sequentially comprises the RT Fingers/Palm, Thumb/X, Linker, and endonuclease-like domains; this rigid core is shifted towards the N-domain of Prp8 in the pre-B-to-B transition, with the endonuclease-like domain translocating by up to 25 Å (Fig. 3b). These changes, which make the inter-domain organization of Prp8 more compact, are likely required for pre-mRNA recognition by U5 and U6 snRNAs.

The most drastic structural rearrangement in the pre-B-to-B transition occurs to Brr2, which is translocated by 120–320 Å and as a whole flipped by nearly 180 degrees^6^ (Fig. 3a). In the pre-B complex, Brr2 is connected to the core of the tri-snRNP through three patches of direct interactions: between its NC and the Jab1 domain of Prp8, between its NC and the convex side of three tetratricopeptide repeats (TPR) of Prp6 (residues 272–373), and between its PWI domain and Sad1 (Fig. 3a; Supplementary information, Fig. S9a). In addition, Brr2 also contacts the Linker and the RNaseH-like domains of Prp8. In the B complex,^6^ Brr2 is connected to the core of the tri-snRNP mainly through its NC, which interacts with the N-terminal fragment of Prp4 and the B complex specific protein FBP21 (Fig. 3a; Supplementary information, Fig. S9b). While maintaining its association with the Jab1 domain of Prp8, Brr2 directly contacts the WD40 domain of Smu1 (Fig. 1a, right panel). The translocation of Brr2 in the human pre-B-to-B transition is apparently indispensable for appropriate engagement of the single-stranded U4 snRNA sequences and subsequent remodeling of the human B complex through its ATPase/helicase activity.^20,21^

### Other proteins in the pre-B-to-B transition

**Fig. 4:**
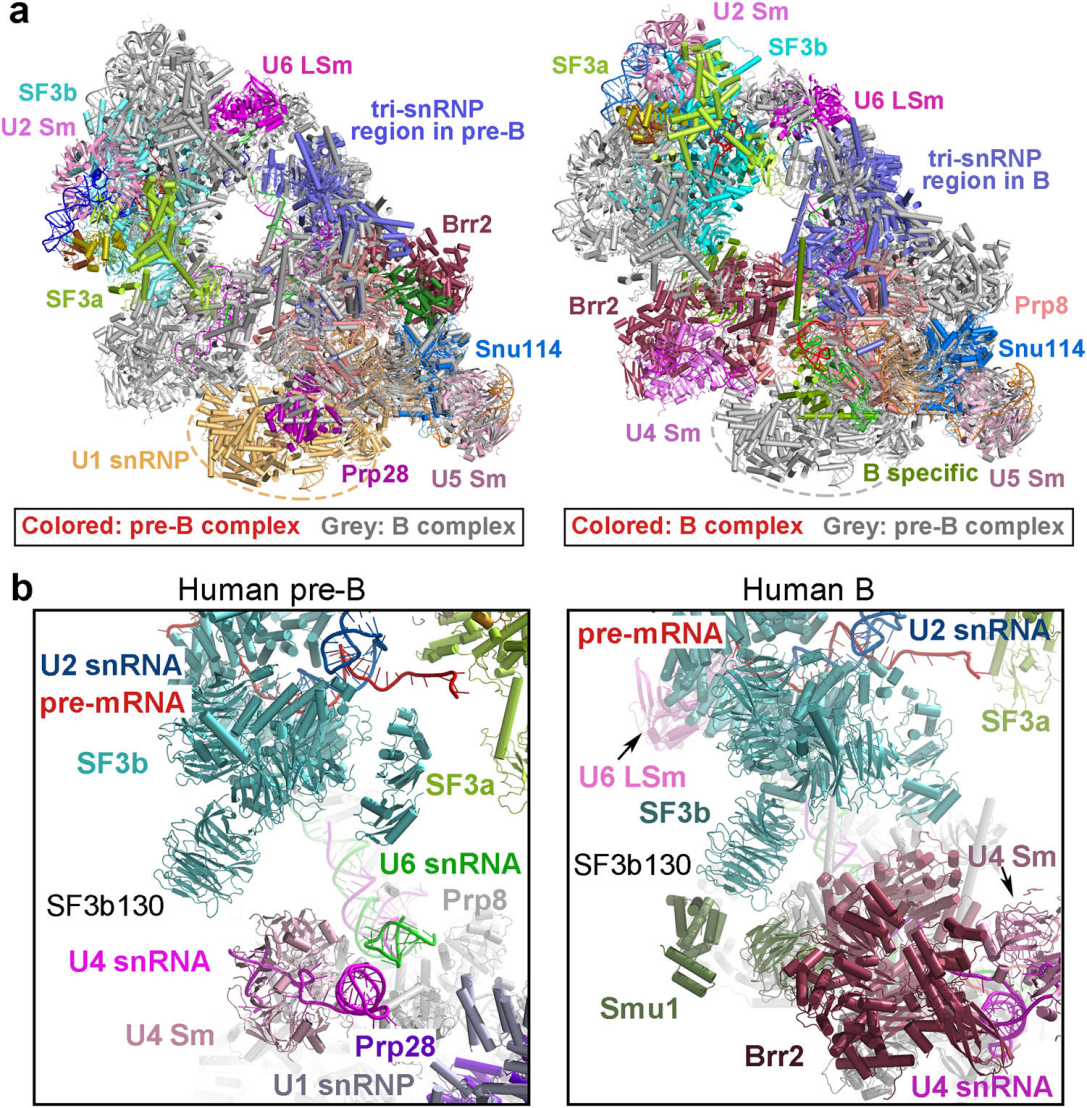
Interactions between the SF3b complex and the U4/U6.U5 tri-snRNP. **a** Overall structural comparison between the human pre-B and B complexes. The two complexes are aligned on the basis of their respective U5 snRNA molecules. In the left panel, the human pre-B complex is color-coded whereas the entire B complex is shown grey. In the right panel, the B complex is color-coded and the pre-B complex is grey. The U6 LSm ring is translocated towards the tri-snRNP in the pre-B-to-B transition. The drastic relocation of Brr2 appears to trigger the translocation of the U4 Sm ring and its associated U4 snRNA sequences. **b** A close-up view on the interactions between the SF3b130 protein of the SF3b complex and the tri-snRNP. In the human pre-B complex (left panel), SF3b130 is close to the U4 Sm ring and its associated U4 snRNA elements. In the human B complex (right panel), SF3b130 directly interacts with the N-terminal α-helical domain of Smu1, whereas Brr2 binds the C-terminal WD40 domain of Smu1

In the pre-B-to-B transition, the vast majority of the protein components in the U4/U6.U5 tri-snRNP and the U2 snRNP undergo marked positional shifts. In the tri-snRNP, Prp3, Prp4, Prp31, Prp6, and Snu13 are each translocated by 10–40 Å, although their relative orientation with each other has remained largely unchanged in the transition (Fig. 3c; Supplementary information, Fig. S9c, d). The C-terminal fragment of Prp3 is shifted by about 25 Å, and the TPR repeats of Prp6 are translocated by approximately 35 Å. Some of the structural changes can be rationalized as a direct consequence of the translocation of Brr2 (Supplementary information, Fig. S9c, d). The relocation of Brr2 from one corner of the tri-snRNP tetrahedron to another results in the translocation of the U4 Sm ring and its associated U4 snRNA sequences farther away from the center of the tri-snRNP (Fig. 4a). In the pre-B complex, the WD40 domain of SF3b130 is located close to the U4 Sm ring (Fig. 4b, left panel). In the B complex,^6^ the WD40 domain of SF3b130 directly binds the N-terminal α-helical domain of Smu1 and Brr2 interacts with the C-terminal WD40 domain of Smu1 (Fig. 4b, right panel). The entire SF3a and SF3b complexes are shifted upward away from the tri-snRNP; their relocation pushes the U6 LSm ring closer towards the tri-snRNP (Fig. 4a).

### Differences of the human and *S. cerevisiae* pre-B-to-B transitions

**Fig. 5:**
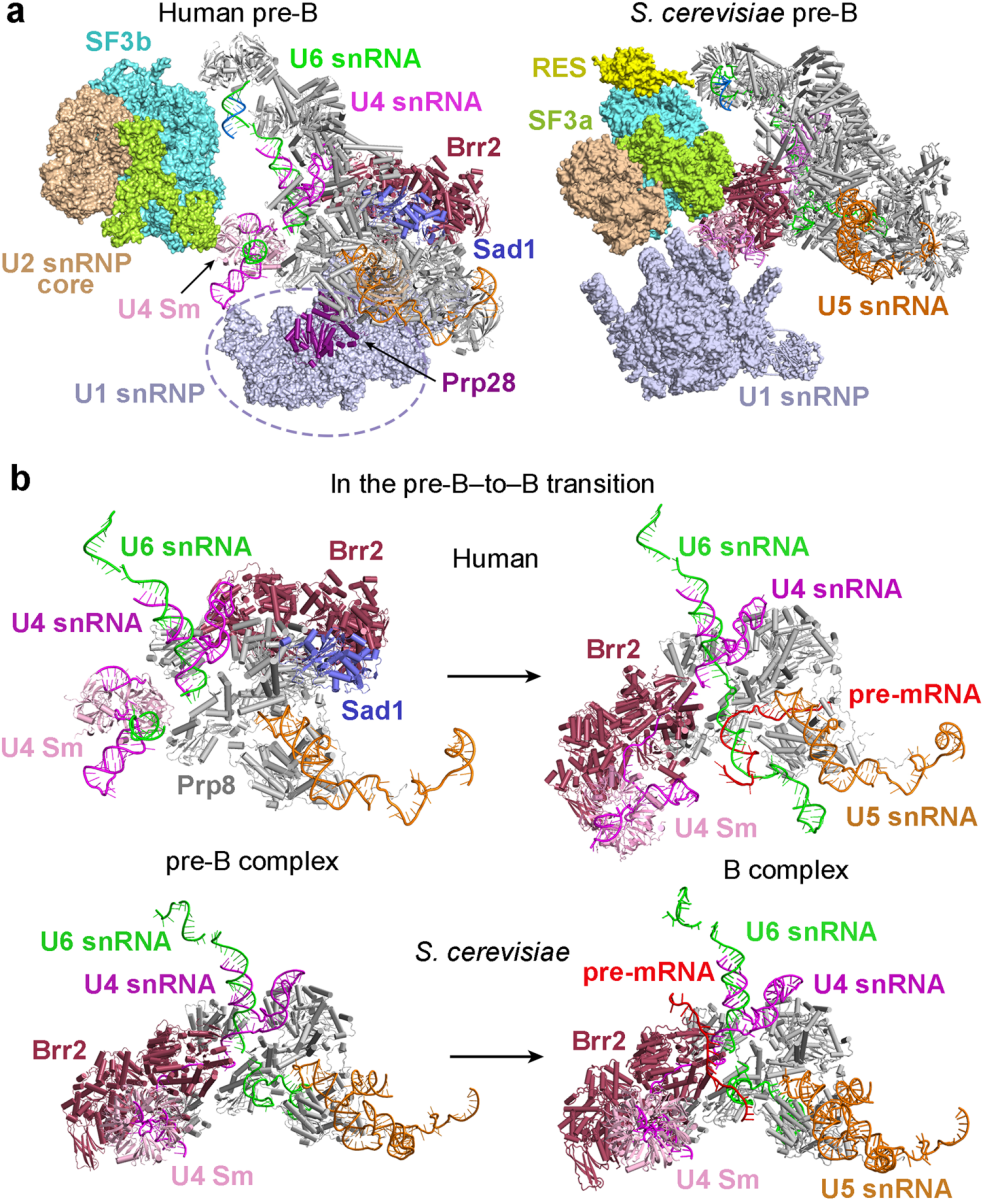
Structural comparison of the human and *S. cerevisiae* pre-B-to-B transitions. **a** Comparison of the overall structures between the human and *S. cerevisiae* pre-B complexes. Compared to *S. cerevisiae,*^14^ the overall appearance of the human pre-B complex is quite different, and the location of U1 snRNP relevant to tri-snRNP has shifted drastically. The retention and splicing (RES) complex is present in the *S. cerevisiae* pre-B and B complexes, but not in the human complexes. **b** Comparison of the RNA elements in the human and *S. cerevisiae* pre-B-to-B transitions. Loop I of U5 snRNA remains unoccupied in the human pre-B complex but forms a duplex with U6 snRNA in the *S. cerevisiae* pre-B complex.^14^ In the human B complex, the 5’SS and the 5’-exon have already been recognized by U6 snRNA and loop I of U5 snRNA; but in *S. cerevisiae*, the 5’SS is yet to be recognized by U6 snRNA and loop I of U5 snRNA remains engaged with U6 snRNA^14^

Structure determination of the human pre-B complex allows comparison with the yeast pre-B complex^14^ and analysis of differences in the pre-B-to-B transition. Compared to *S. cerevisiae*, the overall organization of the human pre-B complex is quite different; the difference is concentrated in two aspects (Fig. 5a). First, the overall shape of the tri-snRNP region is tetrahedral in the human pre-B complex but triangular in the *S. cerevisiae* complex. This difference is caused by the very different locations of Brr2 in the two complexes; the protein Sad1 is uniquely present in the human pre-B complex to connect Brr2 to the tri-snRNP. Intriguingly, the U4 snRNA has already been loaded into Brr2 in the *S. cerevisiae*, but not human, pre-B complex. Second, the location of U1 snRNP is surprisingly different in the human versus *S. cerevisiae* pre-B complexes. In contrast to that in *S. cerevisiae,*^14^ U1 snRNP in the human pre-B complex is located far away from, and does not directly interact with, U2 snRNP. In addition, the retention and splicing (RES) complex is present in the *S. cerevisiae* pre-B and B complexes, but not in the human complexes. This observation suggests that the RES complex is specifically enriched in the human B-to-B^act^ transition.^40^ Prp28, which drives the pre-B-to-B transition, was clearly identified in the human pre-B complex (Fig. 5a); intriguingly, Prp28 is not present in the corresponding location and remains to be identified in the *S. cerevisiae* pre-B complex. The locational difference of Prp28 between the human and yeast pre-B complexes suggests a major mechanistic variation in the formation of the B complex.

The extent of pre-mRNA engagement in the human pre-B and B complexes exhibits major differences compared to that in the respective *S. cerevisiae* spliceosomes^14^ (Fig. 5b). Loop I of U5 snRNA remains unoccupied in the human pre-B complex but forms a duplex with three consecutive nucleotides of U6 snRNA in the *S. cerevisiae* pre-B complex. Thus U6 snRNA serves as a decoy prior to the arrival of the appropriate 5′-exon-5′SS sequences only in the yeast spliceosome. In the *S. cerevisiae* B complex, the 5′SS is yet to be recognized by U6 snRNA and loop I of U5 snRNA remains engaged with U6 snRNA. However, in the human B complex,^6^ the 5′SS and the 5′-exon have already been recognized by the ACAGA box of U6 snRNA and loop I of U5 snRNA, respectively (Fig. 5b). These differences appear to impose a more stringent regulation on the engagement of pre-mRNA by the human spliceosome.

Careful analysis of the EM density maps reveals unanticipated structural features. A small molecule, interpreted as phosphoinositide 1,2,3,4,5,6-hexaphosphate (IP6), is prominently located between the N-domain of Prp8 and loop I of U5 snRNA in the EM density maps of the yeast and human spliceosomes (Supplementary information, Fig. S10). In yeast, IP6 is bound in the B^act^ complex^41^ and beyond, but not yet in the B complex.^14,42^ In contrast, a lobe of EM density suggestive of IP6 is uniformly present in the human tri-snRNP and all human spliceosomes including the pre-B complex. Because IP6 appears to play an important role in stabilizing the recognition of U5 snRNA by Prp8, the differences in IP6 binding may suggest variation in regulation of spliceosome assembly and activation between yeast and human.

## Discussion

The pre-B complex represents a distinct state of the assembled spliceosome, preceding the B complex. Of these eight states, four have been structurally captured for the human spliceosome. In this study, we report the cryo-EM structure of the entire human pre-B complex at an average resolution of 5.7 Å. The human pre-B complex was enriched through the use of a cocktail of phosphatase inhibitors^15,16^ and stabilized through chemical crosslinking. The modest resolution is likely a consequence of the highly dynamic conformation of the human pre-B complex, in which U1 and U2 snRNPs are loosely connected to the U4/U6.U5 tri-snRNP.^12^ Our structure of the human pre-B complex confirms its biochemical identification^12^ and allows visualization of its detailed structural features. As anticipated, the pre-mRNA is yet to be engaged by loop I of U5 snRNA and U1 snRNP remains associated only in the pre-B complex.

**Fig. 6:**
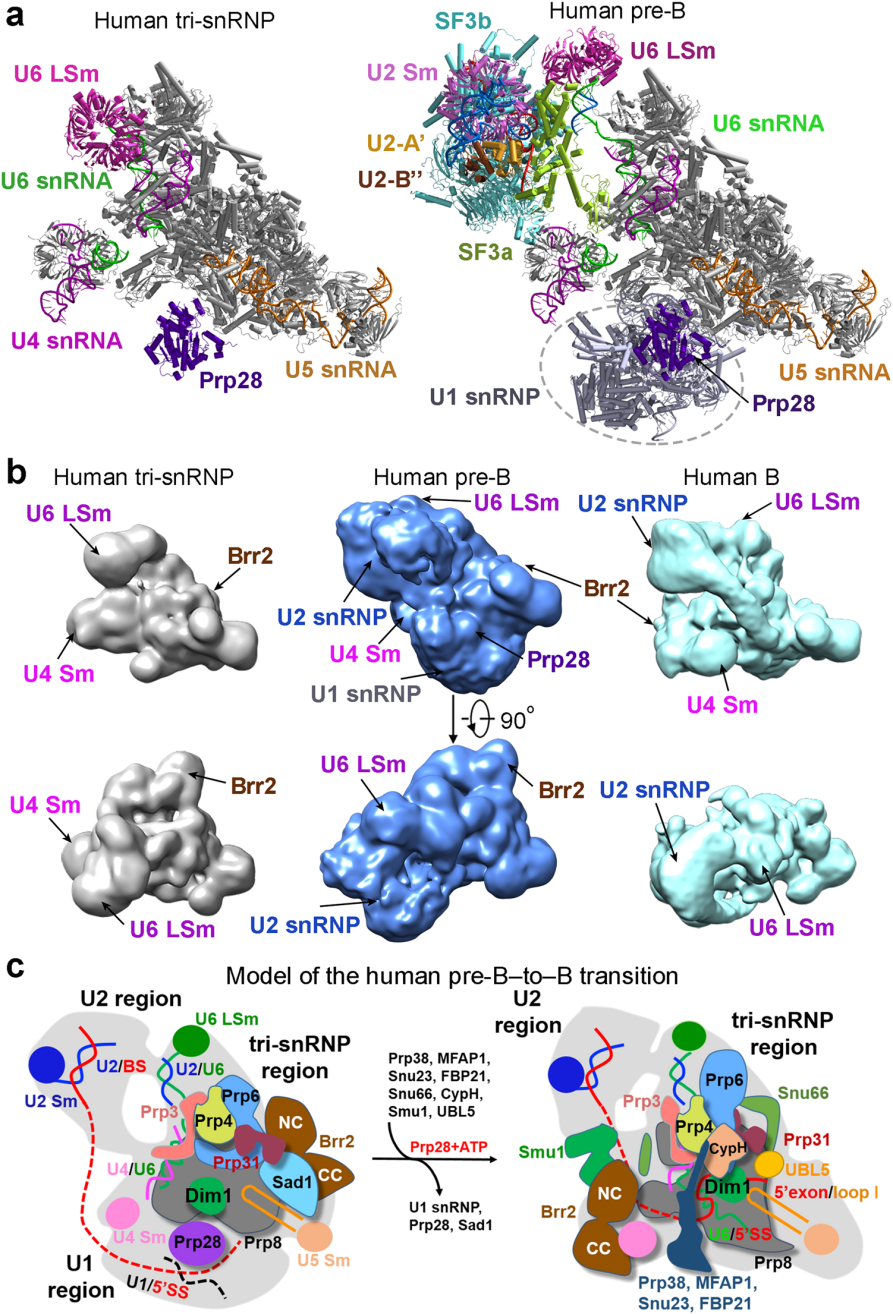
Structural rearrangement from the human U4/U6.U5 tri-snRNP to the human pre-B and B complexes. **a** Comparison of the structures between the human U4/U6.U5 tri-snRNP^13^ (left panel) and the human pre-B complex (right panel). U1 and U2 snRNPs have been recruited into the pre-B complex. **b** Comparison of the EM density maps reveals global differences among the tri-snRNP (left panels), the pre-B complex (middle panels), and the B complex (right panels). These three complexes are oriented identically with respect to the core components of the U4/U6.U5 tri-snRNP. Compared to the tri-snRNP, the human pre-B complex contains two additional large lobes of EM density: one assigned to U1 snRNP and the other to U2 snRNP. The heptameric LSm ring undergoes a positional shift from the tri-snRNP to the pre-B complex. Compared to the pre-B complex (middle panels), the EM density lobe assigned to U1 snRNP is absent in the B complex (right panels). The EM density for U2 snRNP and U6 LSm ring exhibits apparent differences. **c** A cartoon diagram of the human pre-B-to-B transition trigged by the RNA-dependent ATPase/helicase Prp28. One of the most notable changes in this transition is the engagement of pre-mRNA by both U5 and U6 snRNA

The structure of the human pre-B spliceosome allows direct comparison with the human tri-snRNP^13^ (Fig. 6a). The association of the A complex (which comprises U1 and U2 snRNPs and the pre-mRNA) with the tri-snRNP triggers the translocation and rearrangement of a few components in the tri-snRNP. The most notable change is the formation of the U2/U6 duplex in the pre-B complex, which results in the translocation of the nearby RNA elements and the associated heptameric LSm ring (Fig. 6a, b). In the A complex, the 5′SS of the pre-mRNA is presumably recognized by the complementary U1 snRNA sequences.^43–45^ Our current EM map, however, does not have the required quality or resolution for conclusive identification of the individual components of U1 snRNP. This is likely due to the dynamic nature within U1 snRNP as well as its flexible connection to the tri-snRNP.

In this study, we also report the 3.8-Å cryo-EM structure of the entire human B complex, which had been elucidated at a somewhat lower resolution.^6^ Although the key features observed in our structure are similar to those reported previously,^6^ improved resolution allows identification of previously unrecognized structural features. For example, the B-specific protein UBL5 was unambiguously identified in our B complex structure; UBL5 is located close to the active site in the heart of the spliceosome and plays an important role in splicing. Complex formation between UBL5 and Snu66 is also uniquely observed in our B complex structure. In addition, Snu66 is found to serve as a scaffold in the B complex through stabilization of the Switch loop and the β-finger of Prp8 (Fig. 2c). The improved EM density map also allowed us to assign specific interactions between Prp8 and the 5′SS (Fig. 2e).

The overall structures of the pre-B and B complexes are quite different (Fig. 1a). The pre-B-to-B transition is accompanied not just by the dissociation of U1 snRNP, but also by structural rearrangement of the SF3a and SF3b complexes and most components of the tri-snRNP. These gross differences are nicely illustrated by the overall EM density maps of the human pre-B and the B complexes (Fig. 6b). Only a few components of the rigid core remain largely unchanged in the pre-B-to-B transition. All these belong to the U5 snRNP and include the N-domain of Prp8, Snu114, U5-40 K, U5 Sm ring, and U5 snRNA. The changes of the core domain of Prp8 (RT Fingers/Palm, Thumb/X, Linker, endonuclease-like) are also considerably less prominent than those of other components of the spliceosome. This analysis again identifies U5 snRNP as the central scaffold of the assembled spliceosomes.^36,37^ Except these few components, all other proteins and RNA elements have undergone marked changes. This is in sharp contrast to the *S. cerevisiae* case, where most protein components of the tri-snRNP remain unchanged in the pre-B-to-B transition.^14^ The transition from the pre-B to the B complex is driven by Prp28,^17,19^ which is only present in the pre-B, but not the B, complex (Fig. 6c). U1 snRNP and Sad1 are dissociated from the pre-B and at least eight proteins are recruited to form the B complex. Along the way, Prp28 itself is dissociated and Brr2 is translocated to the correct location for the next phase of spliceosome remodeling.

## Materials and methods

### In vitro splicing reaction

Uniformly m7G(5’)ppp(5′)-capped MINX pre-mRNA was in vitro synthesized using the T7 runoff transcription method. The pre-mRNA contains two exons (60 nucleotides and 46 nucleotides in length) that are separated by a 120-nucleotide intron, and three tandem MS2 binding sites that are positioned at the 3′-end of the 3′-exon. Nuclear extract was prepared from HeLa S3 cells when the cell density reaches 1.0 × 10^6^ per mL on the basis of a published protocol.^46^ A typical splicing reaction was performed at 30 °C for 45 min in the presence of 15 nM pre-mRNA and 50% (v/v) HeLa nuclear extract diluted by a buffer containing 2 mM ATP, 20 mM creatine phosphate, 3 mM MgCl_2_, 20 mM HEPES-KOH pH 7.9, and 25 mM KCl.

### Use of the phosphatase inhibitor cocktail

Pre-mRNA splicing is regulated by reversible protein phosphorylation.^15,16^ We performed a pilot experiment to examine the impact of the phosphatase inhibitor cocktail (PhosSTOP, Roche) on splicing. One PhosSTOP tablet dissolved in 10 mL buffer is treated as 1 × dilution. The inhibitor cocktail in a wide range of concentration was incubated with the nuclear extract on ice for 1 h prior to the in vitro splicing reaction. Subsequent RT-PCR analysis of the purified RNA revealed slight and complete inhibition of the splicing reaction at 1 × and 2 × dilution of the cocktail, respectively (Supplementary information, Fig. S1a). To ensure complete inhibition, we used 1 × dilution of the cocktail in subsequent splicing assays.

### Purification and crosslinking of the spliceosomal complexes

The spliceosomal complexes were in vitro assembled using a typical splicing reaction on the MINX pre-mRNA and purified by the MS2-MBP affinity chromatography. Specifically, the phosphatase inhibitor cocktail PhosSTOP at a final 1 × dilution was pre-incubated with the nuclear extract on ice for 1 h. Pre-mRNA at 15 nM was pre-incubated with 50-fold molar excess of the MS2-MBP fusion protein. After spliceosome formation, the resulting solution was incubated with the amylose resin (NEB) for 2 h at 4 °C. Following extensive washing with the G150 buffer (20 mM HEPES-KOH pH7.9, 150 mM NaCl, 1.5 mM MgCl_2_) in the presence of 1% glycerol, 0.01% NP-40), the spliceosomal complexes were eluted using the G150 buffer supplemented with 20 mM maltose.

For cryo-electron microscopy (cryo-EM) study, the eluted sample was loaded onto a 38.6-mL linear 10%–30% (v/v) glycerol gradient in the G150 buffer supplemented with 0%–0.1% EM-grade glutaraldehyde, and centrifuged at 4 °C for 13.5 h at 25,400 rpm using a SW32 rotor (Beckman Coulter).^47^ Similar to previous description,^9^ the sample after centrifugation was manually fractionated from top to bottom and the total RNA in each fraction was extracted and analyzed on an 8% denaturing polyacrylamide gel (Supplementary information, Fig. S1b). Fractions containing the spliceosomal complexes were pooled and concentrated using a 100-kD cut-off filter unit (Amicon Ultra). Glycerol was removed through dialysis using a 20-kD Side-A-Lyzer (Thermo Scientific) in the G150 buffer.

### EM sample preparation and data collection

Uranyl acetate (2% w/v) was used for negative staining. Briefly, the copper grids supported by a thin layer of carbon film (Zhongjingkeyi Technology Co. Ltd) were glow-discharged. A 4-µL aliquot of the sample was applied onto the grid for 1 min and stored at room temperature. Negatively stained sample was imaged on an FEI Tecnai Spirit Bio TWIN microscope operating at 120 kV to verify the sample quality (Supplementary information, Fig. S1c). The sample was further concentrated and applied to the same grids for cryo-EM specimen preparation. Cryo-EM grids were prepared using Vitrobot Mark IV (FEI Company) at 8 °C and with 100 percent humidity. 4-µL aliquots of the sample were applied to glow-discharged grids for 90 s, blotted for 3 s and plunged into liquid ethane cooled by liquid nitrogen.

Micrographs were collected using a Gatan K2 Summit detector (Gatan Company) mounted on a Titan Krios electron microscope (FEI Company) operating at 300-kV and equipped with a GIF Quantum energy filter (slit width 20 eV) (Supplementary information, Fig. S1d). Micrographs were recorded in the super-resolution mode with a normal magnification of 105,000 × , resulting in a calibrated pixel size of 0.669 Å. Each stack of 32 frames was exposed for 8 s, with an exposing time of 0.25 s per frame. The total dose rate was about 8.2 counts/sec/physical-pixel (~4.7 e^−^/sec/Å^2^) for each stack. AutoEMation was used for the fully automated data collection.^48^ All 32 frames in each stack were aligned and summed using the whole-image motion correction program MotionCor2^49^ and binned to a pixel size of 1.338 Å. The defocus value of each image was set from 0.8 to 1.8 μm and was determined by Gctf.^50^

### Image processing and calculation

1,416,400 particles were auto-picked using the deep-learning program DeepPicker.^51^ The convolutional neural network (CNN) model for particle picking was trained using the previous dataset of the ILS complex from *S. pombe.*^36^ A guided multi-reference classification procedure was applied to the full dataset using the program RELION2.0 ^52,53^ (Supplementary information, Fig. S2). Details of this modified procedure were previously described in the manuscript reporting the cryo-EM structure of the human C complex.^9^ The 3D volumes of the human B and tri-snRNP complexes, and four bad classes were used as initial references (Round 1) (Supplementary information, Fig. S2). These six references were low-pass filtered to 40 Å. To avoid the problem of discarding good particles, we simultaneously performed three parallel multi-reference 3D classifications. After the global classification, particles that belong to the B or the pre-B complex were separately used as the input for follow-up local classification. In each case, the good particles from three parallel runs were merged, and the duplicated particles were removed as described previously.^41^ 492,920 particles (34.8% of the original input) for the B complex and 532,644 particles (37.6% of the original input) for the pre-B complex were selected (Supplementary information, Fig. S2).

A second round (Round 2) of multi-reference local 3D classification was separately performed for the particles that were selected for the B or the pre-B complex with six or five references low-pass filtered, respectively. 2 × binned particles (pixel size: 2.676 Å) were used for the classification (Supplementary information, Fig. S2). For the B complex, particles from the good classes (representing 25.3/30.6/29.3% of the input particles) were combined to yield 180,475 particles (representing 12.7% of the total original particles). These 180,475 particles gave rise to a reconstruction of the human B complex with an overall resolution of 4.54 Å after auto-refinement using unbinned particles (pixel size: 1.338 Å). After that, an additional round (Round 3) of 3D classification was performed. The remaining 180,475 particles were classified with a soft mask on the tri-snRNP core region of the B complex. One class containing 137,853 particles (75.3% of the input particles) yielded a reconstruction at an average resolution of 4.50 Å for tri-snRNP region of the human B complex. These particles were further refined using THUNDER, a cryo-EM 3D reconstruction software.^54^ After global and local search, the resolution was improved to 3.8 Å for the tri-snRNP region (Supplementary information, Fig. S2 & S3a, Table S1).

For the pre-B complex, particles from the good classes (representing 26.7/22.8/25.4% of the input particles) were combined to yield 186,161 particles (representing 13.1% of the total original particles). These 186,161 particles gave rise to a reconstruction of the human pre-B complex with an overall resolution of 6.5 Å after auto-refinement using 2 × binned particles (pixel size: 2.676 Å). These particles were also refined by THUNDER.^54^ After global and local search, the resolution was improved to 5.7 Å for the tri-snRNP region (Supplementary information, Fig. S2 & S3a, Table S1). The U2 snRNP region was very flexible in the pre-B complex. In order to improve the quality of this region, an additional round (Round 3) of 3D classification was performed. One class containing 16,960 particles (1.2% of the total original particles) yielded a reconstruction at an average resolution of 10.0 Å for the entire human pre-B complex, which display better quality of the U2 snRNP region.

In the 5.7-Å map of the pre-B complex, the local resolution reaches 4.0–6.0 Å in the core region of the spliceosome; in the 3.8-Å map of the B complex, the local resolution reaches 3.0–4.0 Å in the core region of the spliceosome (Supplementary information, Fig. S3b). The angular distributions of the particles used for the final reconstruction of both human pre-B and B complexes are reasonable (Supplementary information, Fig. S3c, d), and the refinement of the atomic coordinates did not suffer from severe over-fitting (Supplementary information, Fig. S3e). The resulting density maps display clear features for the secondary structural elements of the human pre-B and B complex in the core region. The RNA elements and their interacting proteins are also reasonably well defined by the EM density maps.

Reported resolutions were calculated on the basis of the FSC 0.143 criterion, and the FSC curves were corrected with high-resolution noise substitution methods.^55^ Prior to visualization, all density maps were corrected for the modulation transfer function (MTF) of the detector, and then sharpened by applying a negative B-factor that was estimated using automated procedures.^56^ Local resolution variations were estimated using ResMap.^57^

### Model Building and refinement

Due to a wide range of resolution limits for the various regions of the human spliceosomal pre-B and B complexes, we combined homology modeling and rigid docking of components with known structures to generate the atomic models (Supplementary information, Tables S2 & S3). Identification and docking of the components of the human pre-B and B complexes were facilitated by the structures of the human tri-snRNP, B, B^act^ and C^*^ complexes^6,10,13,58,59^ and the *S. cerevisiae* tri-snRNP and B complex.^34,42,60^ The protein components that were derived from known structures of the protein data bank (PDB) are summarized in Table S2 & S3. These structures were docked into the density map using Coot^61^ and fitted into density using CHIMERA.^62^

For the human B complex, the atomic coordinates of the U2 snRNP and U5 snRNP from the human C^*^ and B^act^ complexes^10,59^ were directly docked into the 3.8 Å density maps, the U5 snRNA and U5 snRNP proteins Prp8, Snu114 and Dim1 were manually adjusted using Coot.^61^ Assignment and model building of the duplex between the 5′-splice site (5′SS) and U6 snRNA and the duplex between U4 and U6 snRNA was greatly aided by the structure of the human C^*^ and B complexes and the structure of yeast tri-snRNP.^6,10,34^ Docking and homology model building of the structures of the tri-snRNP specific proteins was guided by the yeast tri-snRNP,^34^ and the human tri-snRNP and B complex.^6,13^ Docking the structures of the B specific proteins was guided by both the yeast and the human B complexes.^6,42^ The N-terminal HIND motif of Snu66 is located close to the 5′-exon; this information facilitated the identification of the UBL5/HIND complex in the B complex. The crystal structure of the human UBL5/HIND complex^32^ was docked into the EM density near the 5′-exon; this assignment is fully consistent with the reported function of UBL5 in splice-site selection and alternative splicing.^31,32^

For the pre-B complex, the atomic coordinates derived from the human B complex in this study was docked in the 5.7 Å density map of pre-B complex. Crystal structure of Prp28 and Sad1 was docked into the density of the pre-B complex according the cryo-EM structure of human tri-snRNP.^13^ The cryo-EM structure of U1 snRNP^63^ was docked into the bulky density lobe near the ATPase/helicase Prp28. The side chains in the protein components of the pre-B complex were removed due to lack of side chain features.

The final overall models of the pre-B and B complexes were refined against the 5.7-Å map of the pre-B complex and the 3.8-Å map of the B complex, respectively, using REFMAC in reciprocal space^64^ and secondary structure restraints that were generated by ProSMART.^65^ Overfitting of the model of B complex was monitored by refining the model in one of the two independent maps from the gold-standard refinement approach, and testing the refined model against the other map^66^ (Supplementary information, Fig. S3e). The structures of the human pre-B and B complexes were validated through examination of the Molprobity scores and statistics of the Ramachandran plots (Supplementary information, Table S1). Molprobity scores were calculated as described.^67^

### Accession code

The atomic coordinates for the human pre-B and B complexes have been deposited in the Protein Data Bank with the accession code 6AH0 and 6AHD, respectively. The EM maps for the human pre-B and B complexes have been deposited in EMDB with the accession codes EMD-9621 and EMD-9624, respectively.

## Electronic supplementary material

Supplementary information, Table S1

Supplementary information, Table S2

Supplementary information, Table S3

Supplementary information, Figure S1

Supplementary information, Figure S2

Supplementary information, Figure S3

Supplementary information, Figure S4

Supplementary information, Figure S5

Supplementary information, Figure S6

Supplementary information, Figure S7

Supplementary information, Figure S8

Supplementary information, Figure S9

Supplementary information, Figure S10
