# Supplementary material for: Structures of the human pre-catalytic spliceosome and its precursor spliceosome
Source: Cell Res. 2018 Oct 12;28(12):1129–40. doi: 10.1038/s41422-018-0094-7 (PMC6274647; doi:10.1038/s41422-018-0094-7)
Supplement: Supplementary file 1 — Supplementary information, Table S1 [file 41422_2018_94_MOESM1_ESM.pdf]

**Table S1. Cryo-EM data collection and refinement statistics.**

| | |
|---|---|
| **Data collection** | |
| EM equipment | FEI Titan Krios |
| Voltage (kV) | 300 |
| Detector | Gatan K2 |
| Pixel size (Å) | 1.338 |
| Electron dose (e-/Å$^2$) | 45.6 |
| Defocus range (μm) | 0.8~1.8 |
| **Reconstruction (pre-B/B complex)** | |
| Software | RELION 2.0/THUNDER |
| Number of used Particles | 186,162/137,853 |
| Accuracy of rotation (°) | 1.09/0.79 |
| Accuracy of translation (Å) | 0.69/0.52 |
| Final Resolution (Å) | 5.7/3.8 |
| **Model building** | |
| Software | Coot |
| **Refinement (pre-B/B complex)** | |
| Software | Refmac5.8 |
| Map sharpening B-factor (Å$^2$) | -244.4/-140.8 |
| Average Fourier shell correlation | 0.893/0.824 |
| R-factor | 0.25/0.31 |
| **Model composition (pre-B/B complex)** | |
| Protein residues | 16832/14416 |
| RNA nucleotides | 643/550 |
| GTP | 1/1 |
| **Validation (pre-B/B complex)** | |
| R.m.s deviations | |
| Bonds length (Å) | 0.012/0.014 |
| Bonds Angle (°) | 1.661/1.632 |
| Ramachandran plot statistics (%) | |
| Preferred | 92.25/93.64 |
| Allowed | 5.84/3.87 |
| Outlier | 1.90/2.50 |
| Molprobity score | -/3.31 |
