# Supplementary material for: Structures of the human pre-catalytic spliceosome and its precursor spliceosome
Source: Cell Res. 2018 Oct 12;28(12):1129–40. doi: 10.1038/s41422-018-0094-7 (PMC6274647; doi:10.1038/s41422-018-0094-7)
Supplement: Supplementary file 2 — Supplementary information, Table S2 [file 41422_2018_94_MOESM2_ESM.pdf]

Table S2. Summary of model building for the human spliceosomal pre-B complex.

	Molecule Human/*S.pombe*/*S.cere*	Length	Domain/Region	PDB code	Modeling	Resolution (Å)	Chain ID
**U5 snRNP**	U5 snRNA	117	3:116	From B	HM	4.0~5.0	B
	Prp8/Spp42/Prp8	2335	56:662/675:2026/2067:2335		HM	4.0~5.0	A
	Snu114/Cwf10/Snu114	972	112:943		HM	4.0~5.0	C
	Brr2	2136	404:2125		RD	6.0~9.0	D
	U5-40K/Cwf17/-	357	WD40 domain		RD	8.0~10.0	E
	Dim1/Dim1/Dib1	142	Thioredoxin-like		HM	4.0~6.0	O
	SmB,D1,D2,D3,E,F,G	-	Sm fold		RD	6.0~15.0	a-g
**U6 snRNP**	U6 snRNA	107 nt	1:97	From B	RD	4.0~6.0	F
	Lsm2-8	-	Sm fold		RD	9.0~20.0	q-t, x-z
**Pre-mRNA**	Pre-mRNA	-	59 nt	5XJC	RD	4.0~6.0	G
**U4 snRNP**	U4 snRNA	144 nt	1:145	From B	HM	4.0~8.0	I
	SmB,D1,D2,D3,E,F,G	-	Sm fold		RD	5.0~8.0	P-V
**U2 snRNP**	U2 RNA	188 nt	1:47/54:184	5XJC	RD	8.0~20.0	H
	U2-A'/Lea1/Lea1	255	LRR domain	1A9N	RD	20.0~30.0	o
	U2-B"/Msl1/Msl1	225	RRM domain	1A9N	RD	20.0~30.0	p
	SmB,D1,D2,D3,E,F,G	-	Sm fold	4WZJ	RD	20.0~30.0	h-n
	SF3a120/Sap114/Prp21	793	160:294	4DGW	RD	20.0~30.0	u
	SF3a66/Sap62/Prp11	464	92:233		RD	20.0~30.0	v
	SF3a60/Sap61/Prp9	501	1:374/390:463/480:499		RD	20.0~30.0	w
	SF3b155/Sap155/Hsh155	1304	HEAT repeat	5IFE	RD	10.0~20.0	1
	SF3b145/Sap145/Cus1	895	461:600/604:692	5GM6	RD	10.0~20.0	2
	SF3b130/Sap130/Rse1	1217	WD40 domain I/II/III	5IFE	RD	10.0~20.0	3
	SF3b49/Sap49/Hsh49	424	RRM domain I/II	5LSB	RD	10.0~20.0	4
	SF3b14a/p14-like/-	125	RRM domain	5IFE	RD	10.0~20.0	5
	SF3b14b/Ini1/Rds3	110	PHF5 domain	5IFE	RD	10.0~20.0	6
	SF3b10/SF3b10/Ysf3	86	15:80	5IFE	RD	10.0~20.0	7
**Tri-snRNP specific proteins**	Prp3	683	Ferredoxin-like domain	From B	RD	4.0~8.0	J
	Prp4/Cwf3/Syf1	522	WD40 domain		RD	4.0~8.0	K
	Prp31/Cwf7/Snt309	499	Nop domain 52:432		RD	4.0~8.0	L
	Snu13/Cdc5/Cef1	128	5:128		RD	4.0~8.0	M
	Prp6/Cwf4/Clf1	941	NTD; TPR repeat		RD	4.0~8.0	N
	Sad1/Ubp10/Sad1	565	103:565	4MSX	RD	4.0~8.0	W
**ATPase/Helicase**	Prp28	1061	ATP-dependent RNA helicase	4NHO	RD	6.0~15.0	X
**U1 snRNP**	U1 snRNP molecules	-	-	5UZ5	RD	20.0~40.0	-

Under the column labeled "Molecule", proteins from human, *S. pombe*, and *S. cerevisiae* are colored black, red, and green, respectively. If the proteins from all three species have the same name, only a single name in black is indicated. Under the column labeled "Modeling", HM stands for homology modelling; RD stands for rigid docking and refinement.
