# Supplementary material for: Structures of the human pre-catalytic spliceosome and its precursor spliceosome
Source: Cell Res. 2018 Oct 12;28(12):1129–40. doi: 10.1038/s41422-018-0094-7 (PMC6274647; doi:10.1038/s41422-018-0094-7)
Supplement: Supplementary file 4 — Supplementary information, Figure S1 [file 41422_2018_94_MOESM4_ESM.pdf]

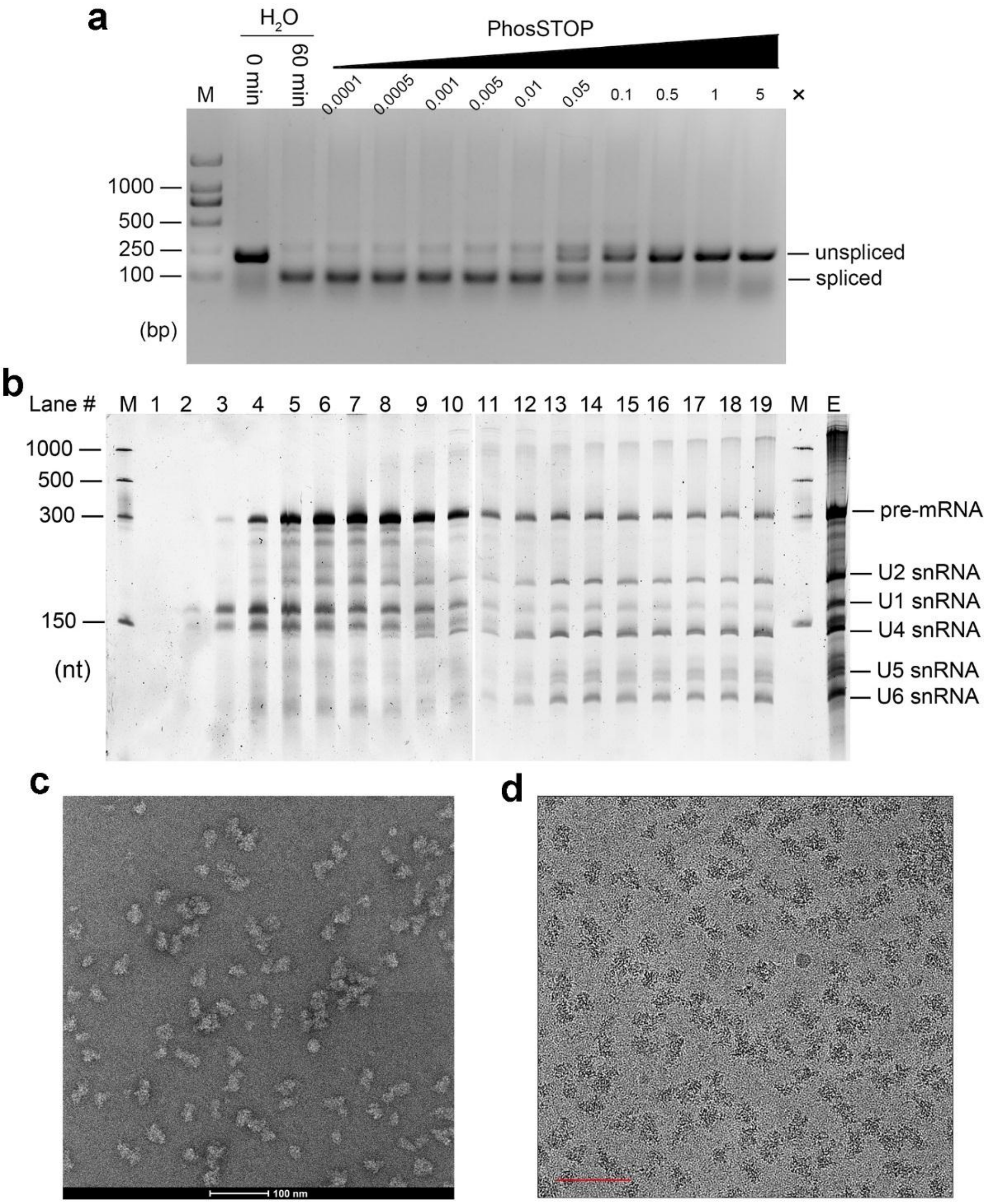

Fig. S1. Purification and EM analysis of the human spliceosomal pre-B and B complexes.

(a) The *in vitro* splicing reaction is suppressed by the phosphatase inhibitor cocktail PhosSTOP (Roche). The splicing reactions were analyzed by RT-PCR, and the results were visualized on an urea PAGE gel here. The 1x label represents the concentration of PhosSTOP where a PhosSTOP tablet is dissolved in 10 mL buffer. (b) Representative results of the *in vitro* splicing reaction under 1x PhosSTOP. The reaction was analyzed on an urea-PAGE gel. (c) A representative electron microscopy (EM) micrograph of the final sample stained by uranyl acetate. Scale bar, 100 nm. (d) A representative cryo-EM micrograph of the final sample. Scale bar, 50 nm.
