# Supplementary material for: Structures of the human pre-catalytic spliceosome and its precursor spliceosome
Source: Cell Res. 2018 Oct 12;28(12):1129–40. doi: 10.1038/s41422-018-0094-7 (PMC6274647; doi:10.1038/s41422-018-0094-7)
Supplement: Supplementary file 5 — Supplementary information, Figure S2 [file 41422_2018_94_MOESM5_ESM.pdf]

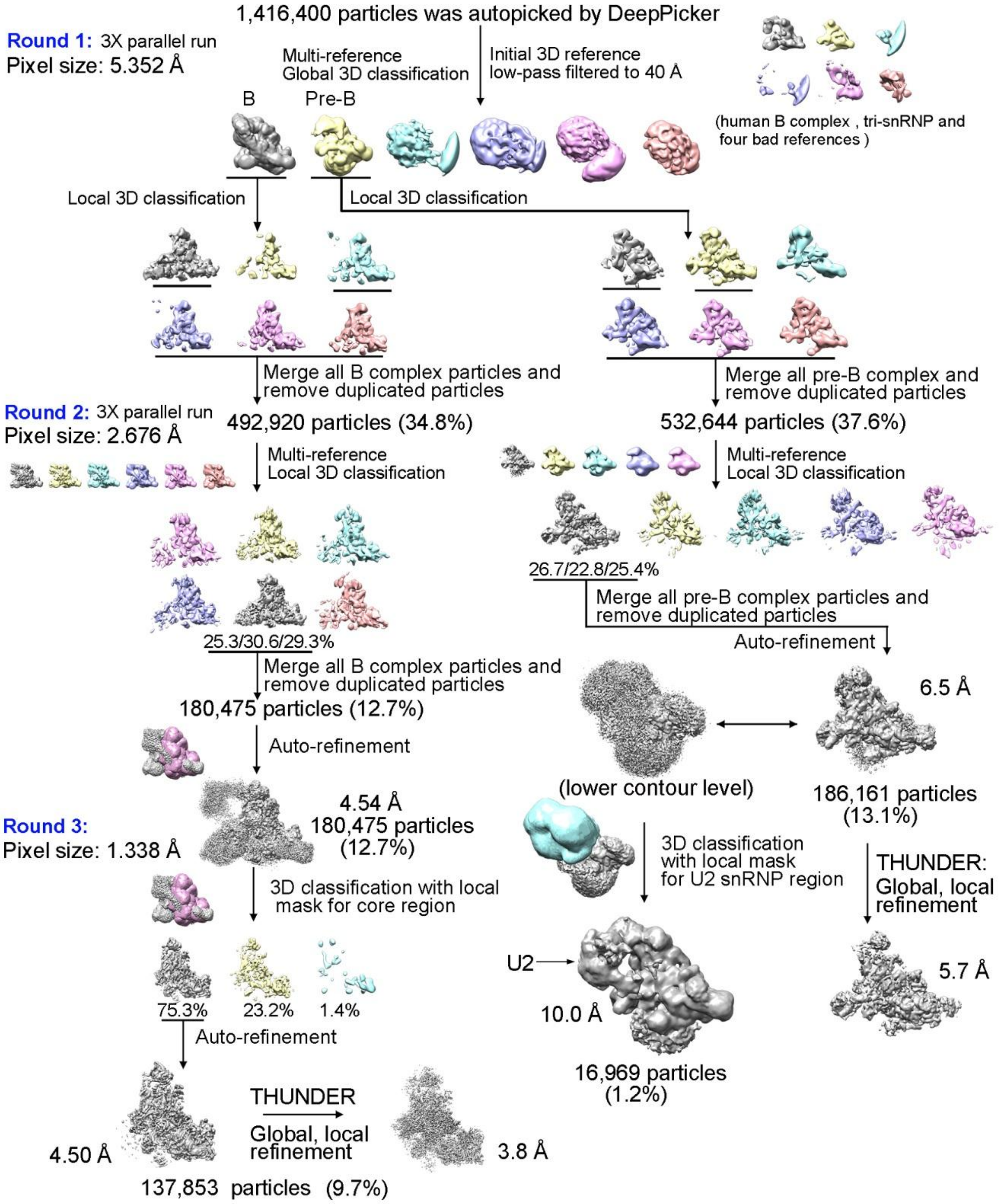


**Fig. S2. A flow chart for the cryo-EM data processing and structure determination of the human spliceosomal pre-B and B complexes**.

On the basis of the FSC value of 0.143, the final reconstruction has an average resolution of 5.7 Å for the pre-B complex and 3.8 Å for the B complex. Please refer to Method Details for the detailed description.
