# Supplementary material for: Structures of the human pre-catalytic spliceosome and its precursor spliceosome
Source: Cell Res. 2018 Oct 12;28(12):1129–40. doi: 10.1038/s41422-018-0094-7 (PMC6274647; doi:10.1038/s41422-018-0094-7)
Supplement: Supplementary file 6 — Supplementary information, Figure S3 [file 41422_2018_94_MOESM6_ESM.pdf]

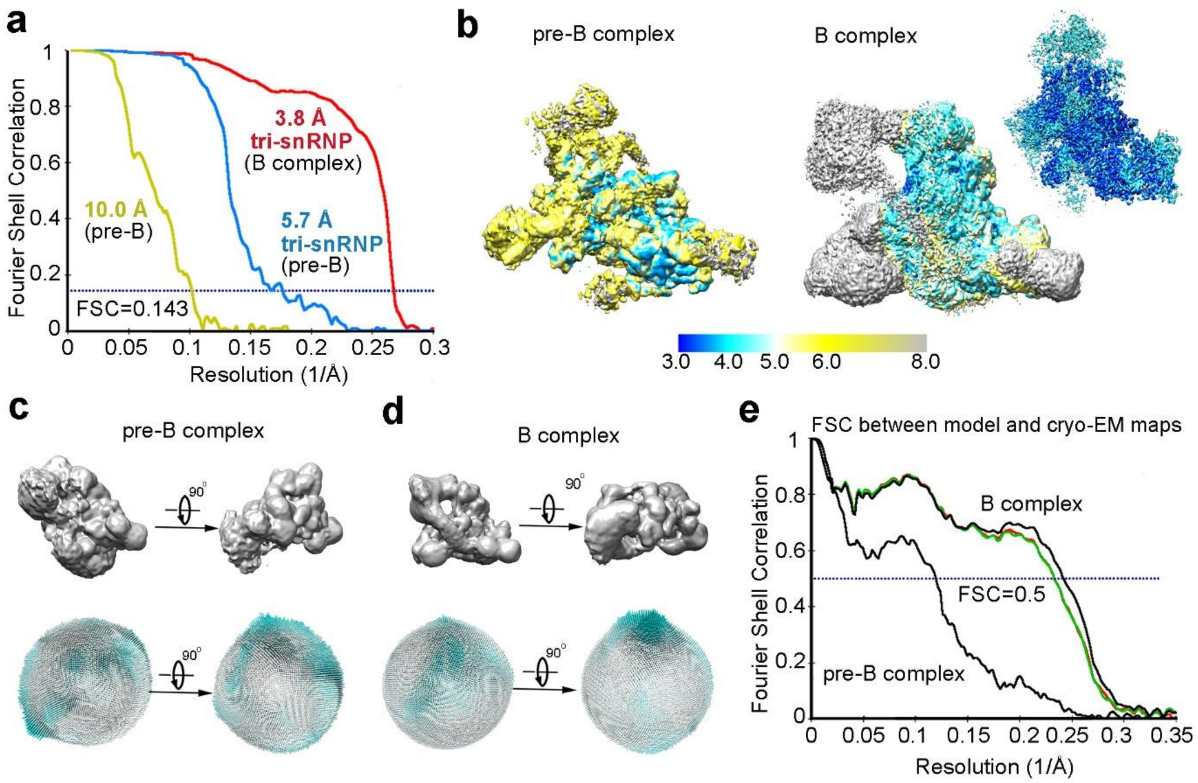

Fig. S3. Cryo-EM analysis of the human spliceosomal pre-B and B complexes.
(a) The average resolutions of the map for the pre-B and B complexes are estimated to be 5.7 and 3.8 Å, respectively, on the basis of the FSC criterion of 0.143. The average resolution for a subset of the pre-B particles with improved EM density for the U2 snRNP region is estimated to be 10.0 Å. (b) The local resolutions are color-coded for different regions of the pre-B complex (left panel) and the B complex (right panel). (c) Angular distribution of the particles used for the reconstruction of the human pre-B complex. Each cylinder represents one view and the height of the cylinder is proportional to the number of particles for that view. (d) Angular distribution of the particles used for the reconstruction of the human B complex. (e) The FSC curves of the final refined model of the human pre-B complex versus the overall map it was refined against (black). The FSC curves of the final refined model of the human B complex versus the overall map it was refined against (black); of the model refined in the first of the two independent maps used for the gold-standard FSC versus that same map (red); and of the model refined in the first of the two independent maps versus the second independent map (green). The generally similar appearances between the red and green curves indicates that the refinement of the atomic coordinates did not suffer from severe over-fitting.
