# Supplementary material for: Structures of the human pre-catalytic spliceosome and its precursor spliceosome
Source: Cell Res. 2018 Oct 12;28(12):1129–40. doi: 10.1038/s41422-018-0094-7 (PMC6274647; doi:10.1038/s41422-018-0094-7)
Supplement: Supplementary file 7 — Supplementary information, Figure S4 [file 41422_2018_94_MOESM7_ESM.pdf]

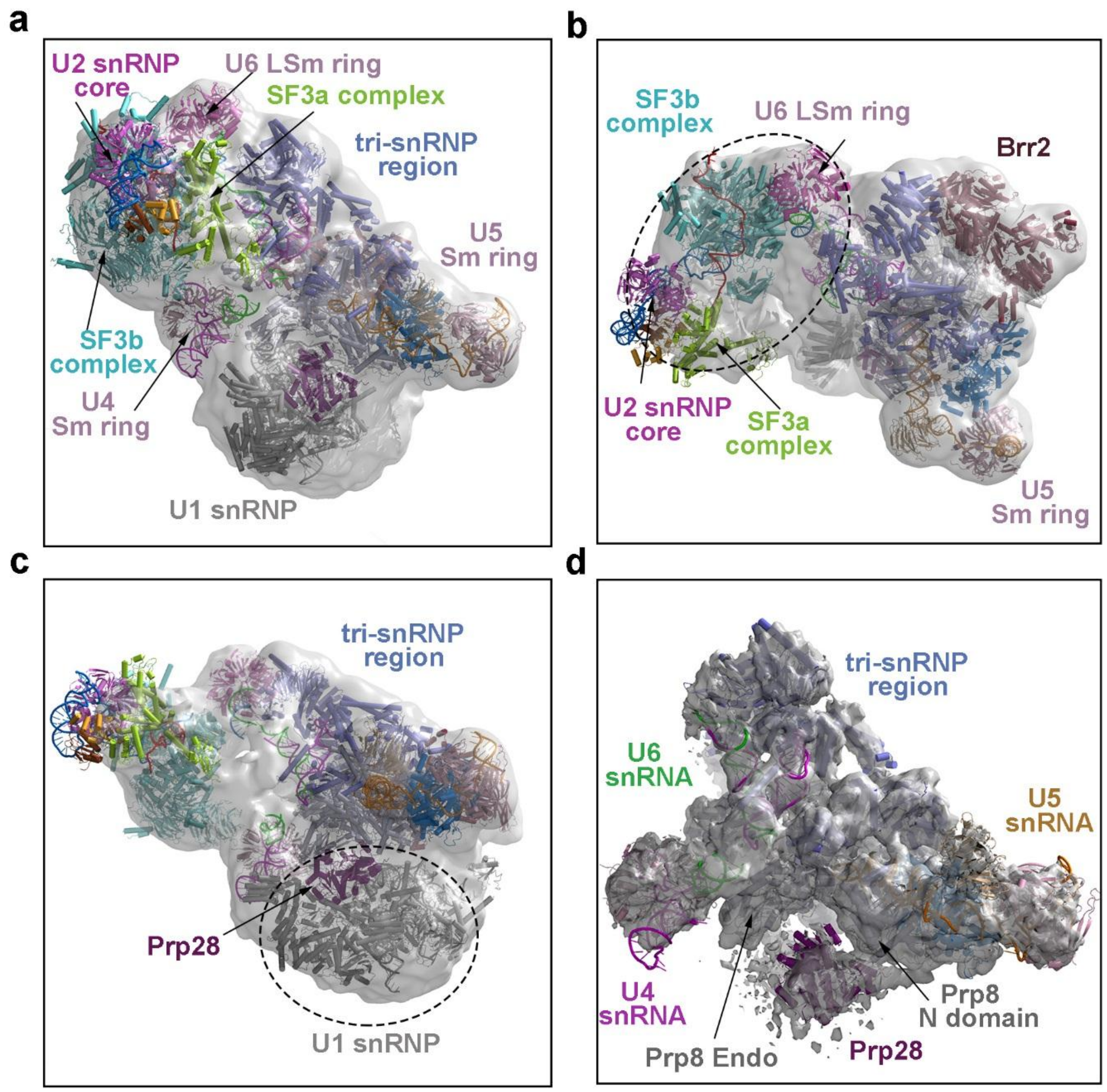

Fig. S4. The cryo-EM density map of the human pre-B complex.
(a) The overall EM density map of the human pre-B complex is low-pass filtered to 20 Å. U2 and U1 snRNPs are flexible and can only be visualized in the low-resolution map. U2 snRNP comprises three parts: the core (U2 snRNA, the Sm ring, and U2-A' and U2-B") (magenta), the SF3a complex (lime), and the SF3b complex (cyan). In this view, U2 snRNP is located in the bulk density in the upper left region of the U4/U6.U5 tri-snRNP. U1 snRNP appears to be located in the region just below the tri-snRNP. (b) A focused view on U2 snRNP. The SF3b complex is connected to the U6 LSm ring and helix II of the U2/U6 snRNA duplex. (c) A focused view on U1 snRNP. A large lobe of EM density is located just below the tri-snRNP. This lobe is flexibly connected to the tri-snRNP and tentatively assigned to U1 snRNP. The RNA-dependent ATPase/helicase Prp28 is located between U1 snRNP and tri-snRNP, ready to unwind the 5'SS/U1 snRNA duplex. (d) The cryo-EM density for the core region of the pre-B complex. The ATPase/helicase Prp28 is connected to the tri-snRNP via the N-domain of Prp8.
