# Supplementary material for: Structures of the human pre-catalytic spliceosome and its precursor spliceosome
Source: Cell Res. 2018 Oct 12;28(12):1129–40. doi: 10.1038/s41422-018-0094-7 (PMC6274647; doi:10.1038/s41422-018-0094-7)
Supplement: Supplementary file 8 — Supplementary information, Figure S5 [file 41422_2018_94_MOESM8_ESM.pdf]

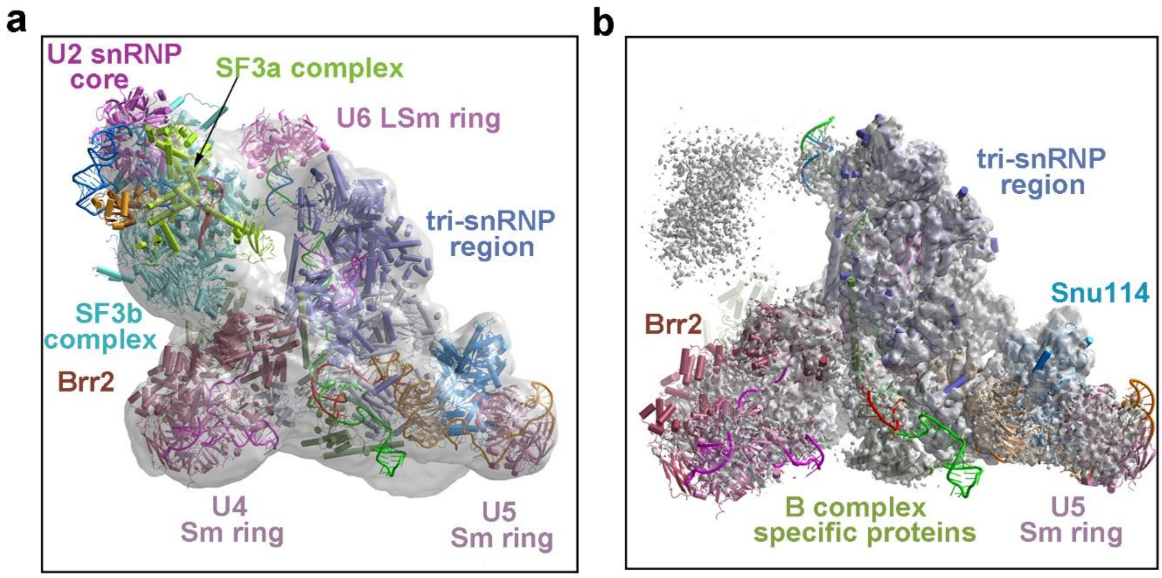

Fig. S5. The cryo-EM density of the human B complex.
(a) The overall EM density map is low-pass filtered to 20 Å. U2 snRNP is flexibly tethered to the tri-snRNP and can only be visualized in the low-resolution map. U2 snRNP comprises three parts: the core (U2 snRNA, the Sm ring, and U2-A' and U2-B") (magenta), the SF3a complex (lime), and the SF3b complex (cyan). These U2 snRNP components are located in the upper left region of the tri-snRNP. Compared to that in the pre-B complex, Brr2 is translocated from the back of the tri-snRNP to the bottom left corner in the B complex. (b) The cryo-EM density of the core region of the B complex. The ATPase/helicase Prp28 is released after its function and no longer present in the B complex. The B complex specific proteins have been recruited to stabilize the overall structure.
