# Supplementary material for: Structures of the human pre-catalytic spliceosome and its precursor spliceosome
Source: Cell Res. 2018 Oct 12;28(12):1129–40. doi: 10.1038/s41422-018-0094-7 (PMC6274647; doi:10.1038/s41422-018-0094-7)
Supplement: Supplementary file 9 — Supplementary information, Figure S6 [file 41422_2018_94_MOESM9_ESM.pdf]

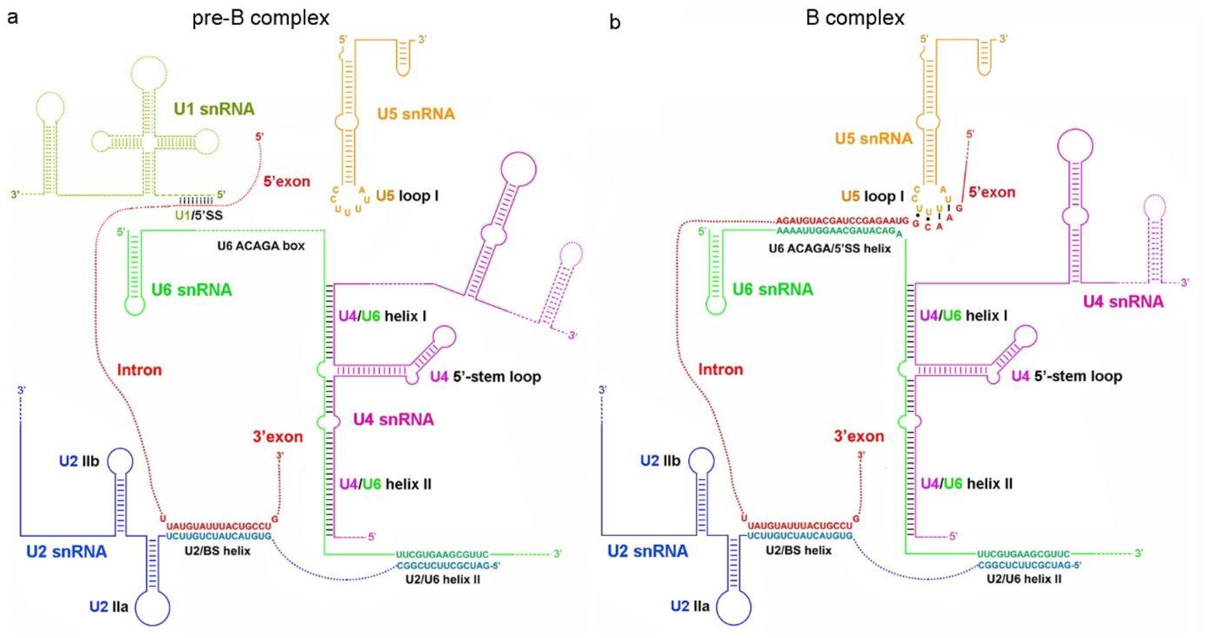

Fig. S6. The two-dimensional RNA diagrams of the human pre-B and B complexes.
(a) A schematic diagram of the base-pairing interactions among the RNA elements in the pre-B complex. Loop I of U5 snRNA remains unoccupied; the 5'SS and BPS of the pre-mRNA are recognized by U1 and U2 snRNAs, respectively. (b) A schematic diagram of the base-pairing interactions among the RNA elements in the B complex. Loop I of U5 snRNA is engaged with the 5' exon and the 5'SS forms a duplex with U6 snRNA.
