# Supplementary material for: Structures of the human pre-catalytic spliceosome and its precursor spliceosome
Source: Cell Res. 2018 Oct 12;28(12):1129–40. doi: 10.1038/s41422-018-0094-7 (PMC6274647; doi:10.1038/s41422-018-0094-7)
Supplement: Supplementary file 10 — Supplementary information, Figure S7 [file 41422_2018_94_MOESM10_ESM.pdf]

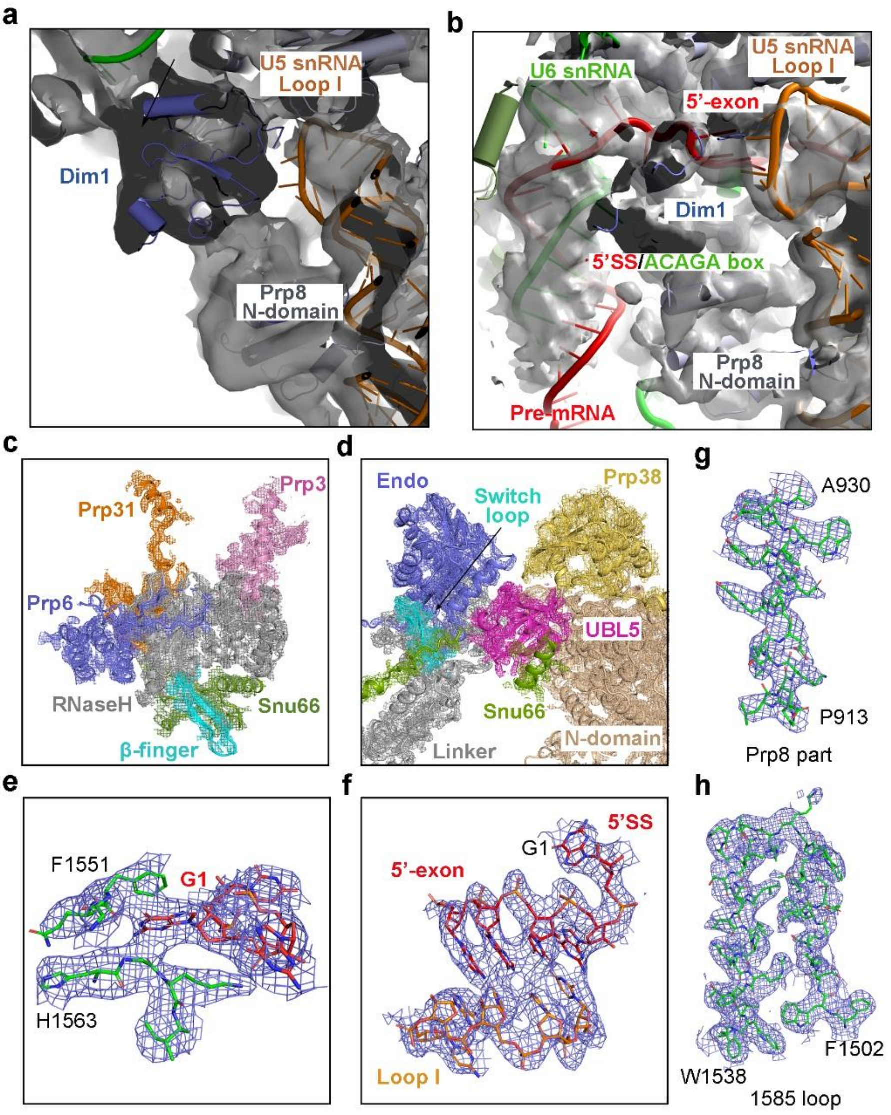

Fig. S7. The EM density maps for the core regions of the human pre-B and B complexes.

(a) A close-up view on the EM density map around the loop I region of U5 snRNA in the human pre-B complex. Dim1 is located close to loop I. In this region, no extra density is present to accommodate the pre-mRNA or U6 snRNA. The U5 loop I remains unoccupied and is available for duplex formation with the 5'-exon of the pre-mRNA. (b) A close-up view on the EM density map around the loop I region of U5 snRNA in the human B complex. Dim1 is still located in the same place close to loop I. In contrast to the pre-B complex, a lobe of density characteristic of an RNA duplex has appeared, suggesting duplex formation between the 5'SS (red) and U6 snRNA (green). Most notably, the 5'-exon is now recruited to loop I. (c) A close-up view on the EM density map around the RNaseH-like domain of Prp8 in the human B complex. (d) A close-up view on the EM density map around UBL5 in the human B complex. (e) A close-up view on the EM density map around the 5'SS in the human B complex. (f) A close-up view on the EM density map around the RNA duplex between the 5'-exon and loop I in the human B complex. (g) Representative EM density of one helix from Prp8 in the human B complex. (h) Representative EM density of the 1585-loop from Prp8 in the human B complex.
