# Supplementary material for: Structures of the human pre-catalytic spliceosome and its precursor spliceosome
Source: Cell Res. 2018 Oct 12;28(12):1129–40. doi: 10.1038/s41422-018-0094-7 (PMC6274647; doi:10.1038/s41422-018-0094-7)
Supplement: Supplementary file 11 — Supplementary information, Figure S8 [file 41422_2018_94_MOESM11_ESM.pdf]

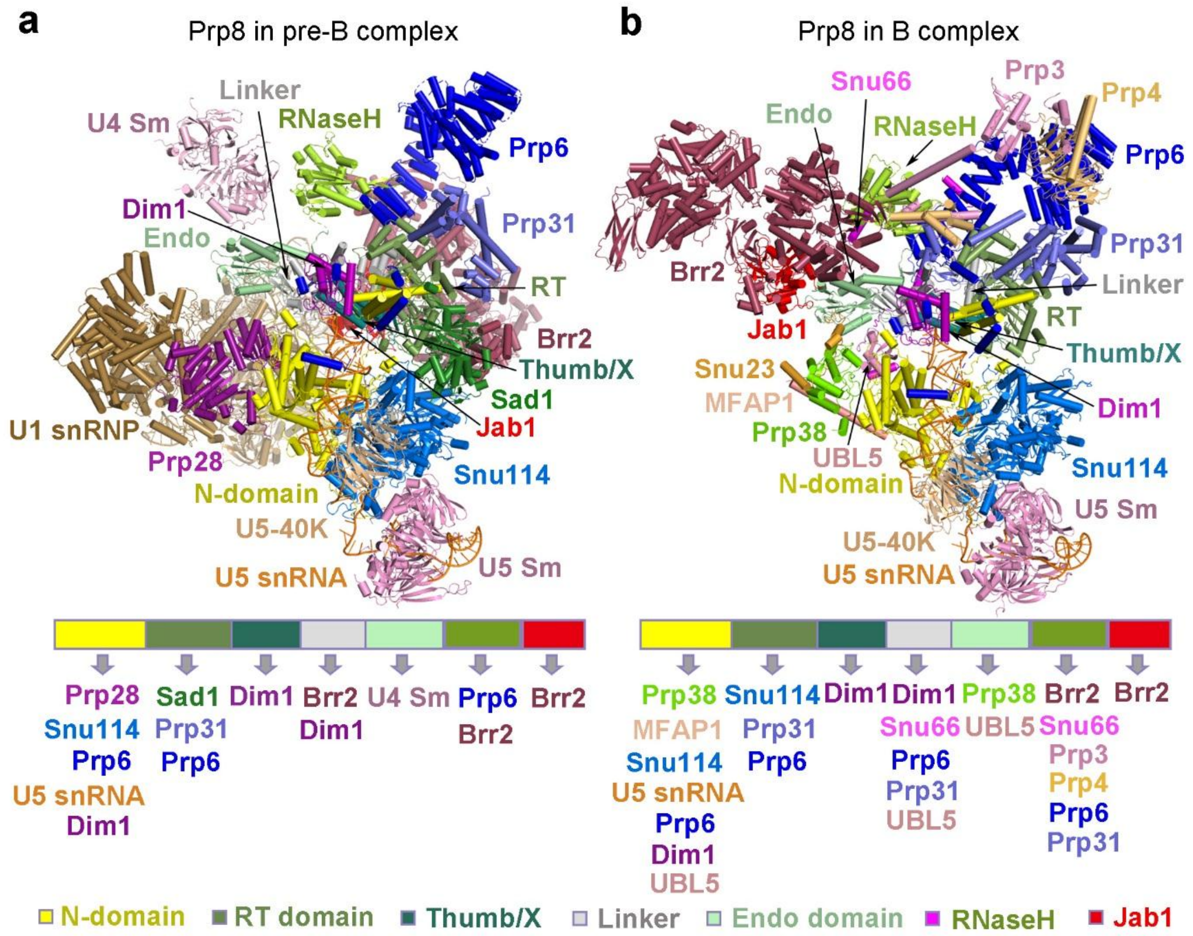

Fig. S8. Structural comparison of Prp8 between the human pre-B and the B complexes. (a) Structure of Prp8 in the human pre-B complex. Some of the Prp8-binding proteins are shown. The seven domains of Prp8 are color-coded and their interacting proteins are tabulated below the structure. (b) Structure of Prp8 in the human B complex. Structural comparison of Prp8 in panels A and B reveals major differences in the Jab1 and RNaseH-like domains and to a lesser extent in the core of Prp8 (endonuclease-like, Linker, Thumb/X, RT Fingers/Palm) and a portion of the N-domain (residues 663-798).
