# Supplementary material for: Structures of the human pre-catalytic spliceosome and its precursor spliceosome
Source: Cell Res. 2018 Oct 12;28(12):1129–40. doi: 10.1038/s41422-018-0094-7 (PMC6274647; doi:10.1038/s41422-018-0094-7)
Supplement: Supplementary file 12 — Supplementary information, Figure S9 [file 41422_2018_94_MOESM12_ESM.pdf]

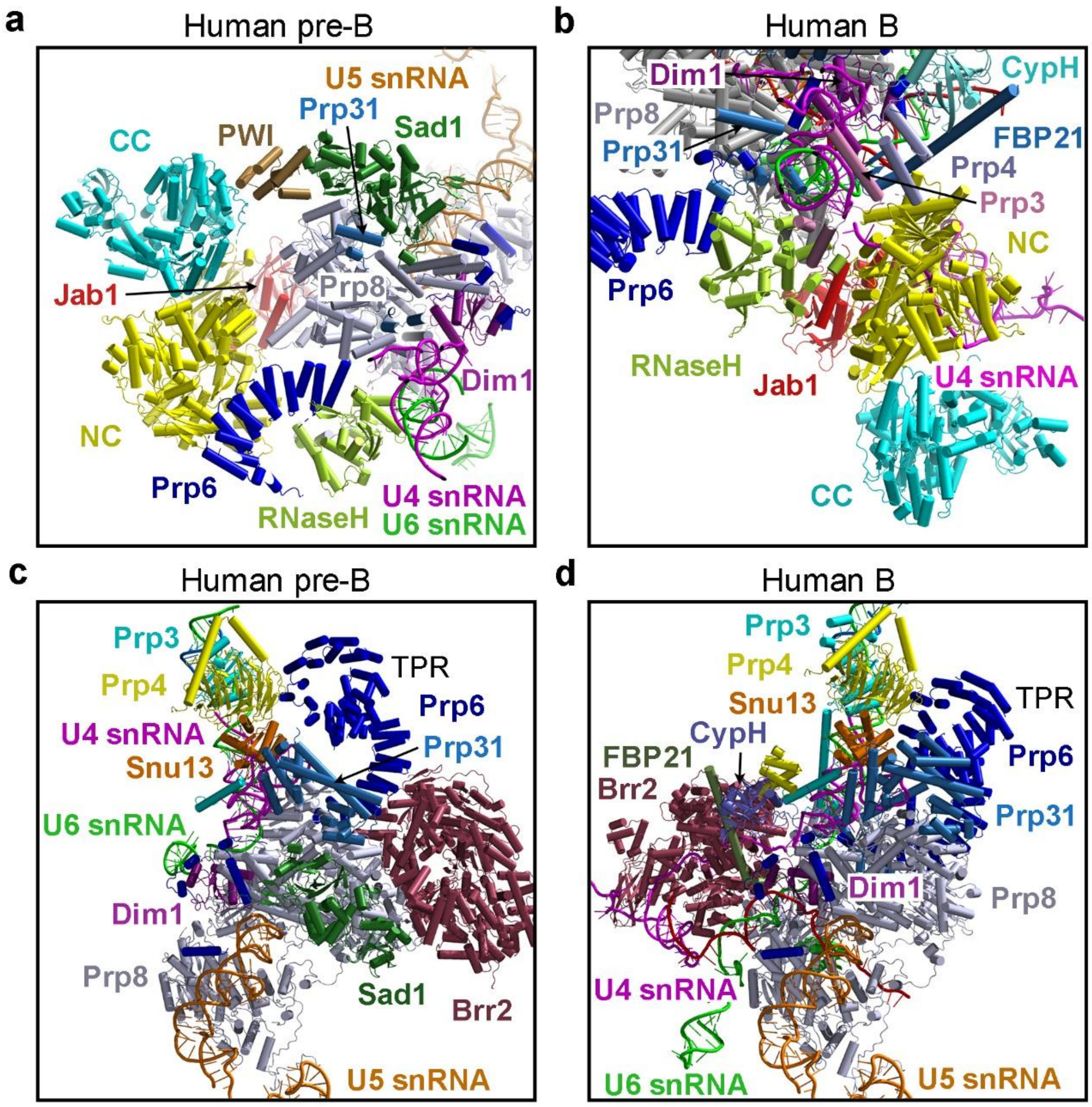

Fig. S9. Structural comparison of components of the tri-snRNP between the human pre-B and B complexes.

(a) Structure of Brr2 in the human pre-B complex. The NC and CC of Brr2 are colored yellow and cyan, respectively. Neighboring proteins are shown. (b) Structure of Brr2 in the human pre-B complex. (c) Structure of select protein components of the U4/U6.U5 tri-snRNP in the human pre-B complex. (d) Structure of select protein components of the U4/U6.U5 tri-snRNP in the human B complex. Comparison of panels C and D reveals marked positional shifts in the protein components. Prp3, Prp4, Prp31, Prp6, and Snu13 are translocated by 10-40 Å in the transition. Sad1 is located between Brr2 and Prp8 in the pre-B complex but is absent in the B complex.
