# Supplementary material for: Structures of the human pre-catalytic spliceosome and its precursor spliceosome
Source: Cell Res. 2018 Oct 12;28(12):1129–40. doi: 10.1038/s41422-018-0094-7 (PMC6274647; doi:10.1038/s41422-018-0094-7)
Supplement: Supplementary file 13 — Supplementary information, Figure S10 [file 41422_2018_94_MOESM13_ESM.pdf]

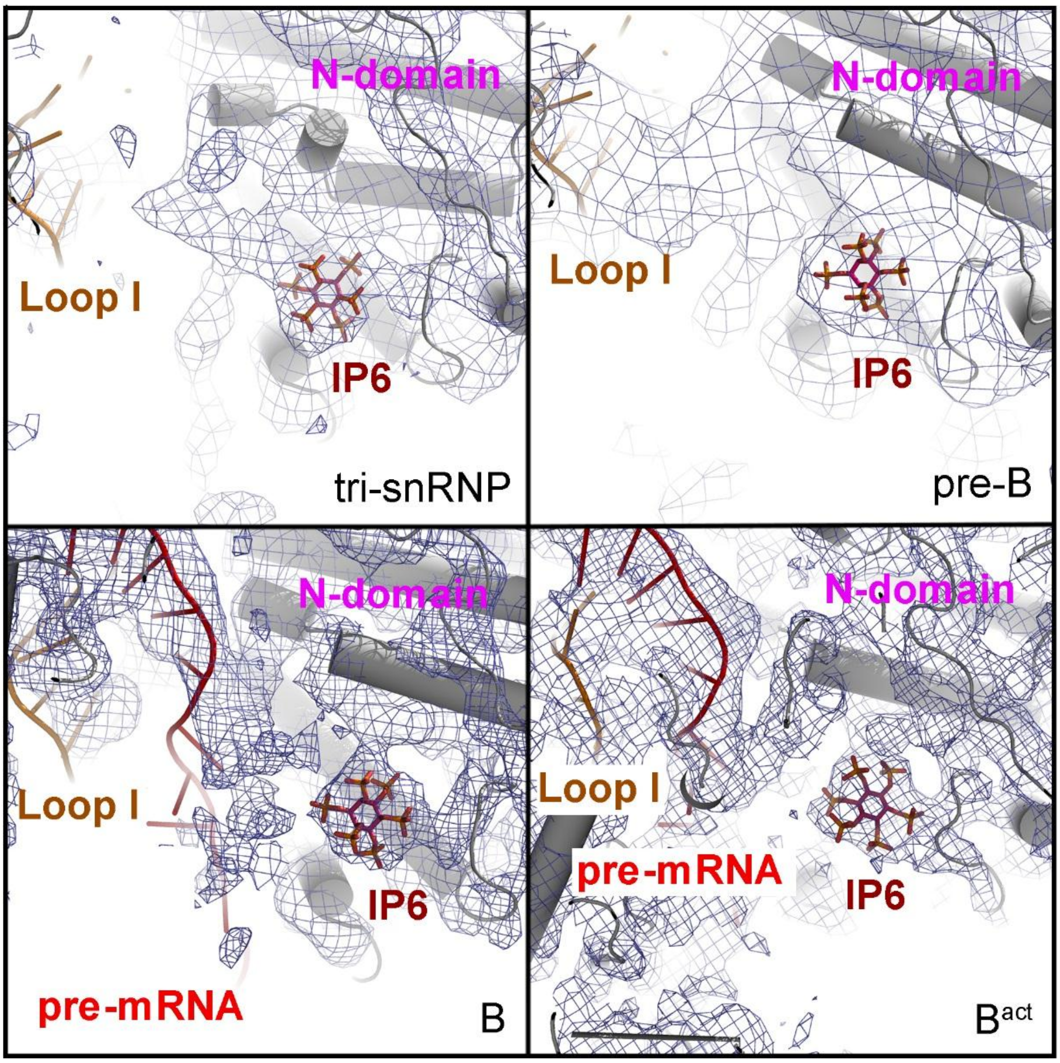

Fig. S10. Presence of a small molecule in the structure of the human tri-snRNP, pre-B, B, and B^{act} complexes.

This small molecule, identified as phosphoinositide 1,2,3,4,5,6-hexaphosphate (IP6), stabilizes the recognition of U5 snRNA by the N-domain of Prp8.
